# Screening for hypertension in adults: protocol for evidence reviews to inform a Canadian Task Force on Preventive Health Care guideline update

**DOI:** 10.1186/s13643-023-02392-1

**Published:** 2024-01-05

**Authors:** Nicole Shaver, Andrew Beck, Alexandria Bennett, Brenda J. Wilson, Chantelle Garritty, Melissa Subnath, Roland Grad, Navindra Persaud, Guylène Thériault, Jennifer Flemming, Brett D. Thombs, John LeBlanc, Janusz Kaczorowski, Peter Liu, Christopher E. Clark, Gregory Traversy, Eva Graham, Janusz Feber, Frans H. H. Leenen, Kamila Premji, Robert Pap, Becky Skidmore, Melissa Brouwers, David Moher, Julian Little

**Affiliations:** 1 School of Epidemiology and Public Health, Faculty of Medicine, University of Ottawa, Ottawa, ON Canada; 2 Division of Community Health and Humanities, Faculty of Medicine, Memorial University of Newfoundland, St. John’s, Canada; 3 Global Health and Guidelines Division, Public Health Agency of Canada, Ottawa, Canada; 4 Department of Family Medicine, McGill University, Montreal, QC Canada; 5 Department of Family and Community Medicine, St. Michael’s Hospital, University of Toronto, Toronto, ON Canada; 6 Department of Medicine, Queen’s University, Kingston, ON Canada; 7 Lady Davis Institute of the Jewish General Hospital, Montreal, QC Canada; 8 Department of Pediatrics, Dalhousie University, Halifax, NS Canada; 9 Department of Family and Emergency Medicine, University of Montreal, Montreal, QC Canada; 10 University of Ottawa Heart Institute, University of Ottawa, Ottawa, ON Canada; 11 Primary Care Research Group, University of Exeter Medical School, Exeter, Devon England; 12 Substance-Related Harms Division, Public Health Agency of Canada, Ottawa, ON Canada; 13 Children’s Hospital of Eastern Ontario, Ottawa, ON Canada; 14 Department of Medicine and Department of Cellular and Molecular Medicine, University of Ottawa, Ottawa, ON Canada; 15 Department of Family Medicine, Faculty of Medicine, University of Ottawa, Ottawa, ON Canada; 16 Schulich School of Medicine and Dentistry, University of Western Ontario, London, ON Canada; 17 Independent Information Specialist, Ottawa, ON Canada; 18 Clinical Epidemiology Program, Ottawa Hospital Research Institute, Ottawa, ON Canada; 19 Department of Family and Community Medicine, University of Toronto, Toronto, Canada; 20 Kingston Health Sciences Centre, Kingston, Canada; 21 Faculty of Medicine, McGill University, Montreal, Canada; 22 Department of Pediatrics, University of Ottawa, Ottawa, Canada

**Keywords:** Systematic review, Overview of reviews, Adults, Guideline, Primary care, Hypertension, Screening, Prediction, Clinically actionable values, Acceptability, Equity

## Abstract

**Purpose:**

To inform updated recommendations by the Canadian Task Force on Preventive Health Care on screening in a primary care setting for hypertension in adults aged 18 years and older. This protocol outlines the scope and methods for a series of systematic reviews and one overview of reviews.

**Methods:**

To evaluate the benefits and harms of screening for hypertension, the Task Force will rely on the relevant key questions from the 2021 United States Preventive Services Task Force systematic review. In addition, a series of reviews will be conducted to identify, appraise, and synthesize the evidence on (1) the association of blood pressure measurement methods and future cardiovascular (CVD)-related outcomes, (2) thresholds for discussions of treatment initiation, and (3) patient acceptability of hypertension screening methods. For the review of blood pressure measurement methods and future CVD-related outcomes, we will perform a de novo review and search MEDLINE, Embase, CENTRAL, and APA PsycInfo for randomized controlled trials, prospective or retrospective cohort studies, nested case–control studies, and within-arm analyses of intervention studies. For the thresholds for discussions of treatment initiation review, we will perform an overview of reviews and update results from a relevant 2019 UK NICE review. We will search MEDLINE, Embase, APA PsycInfo, and Epistemonikos for systematic reviews. For the acceptability review, we will perform a de novo systematic review and search MEDLINE, Embase, and APA PsycInfo for randomized controlled trials, controlled clinical trials, and observational studies with comparison groups. Websites of relevant organizations, gray literature sources, and the reference lists of included studies and reviews will be hand-searched. Title and abstract screening will be completed by two independent reviewers. Full-text screening, data extraction, risk-of-bias assessment, and GRADE (Grading of Recommendations Assessment, Development and Evaluation) will be completed independently by two reviewers. Results from included studies will be synthesized narratively and pooled via meta-analysis when appropriate. The GRADE approach will be used to assess the certainty of evidence for outcomes.

**Discussion:**

The results of the evidence reviews will be used to inform Canadian recommendations on screening for hypertension in adults aged 18 years and older.

**Systematic review registration:**

This protocol is registered on PROSPERO and is available on the Open Science Framework (osf.io/8w4tz).

**Supplementary Information:**

The online version contains supplementary material available at 10.1186/s13643-023-02392-1.

## Background

### Definition

Blood pressure is a measure of the force of blood pushing against arterial walls. High blood pressure, or hypertension, is a common condition in which the blood vessels sustain persistently raised pressure [1, 2]. Large-scale population-based studies have found that the relationship between blood pressure and risk of cardiovascular disease is continuous and follows a decreasing gradient with no apparent threshold, at least down to a blood pressure of 115/75 mm Hg [3, 4]. Hypertension is often first observed through office-based screening and then diagnosed with follow-up blood pressure measurements. In Canada, the 2020 Hypertension Canada guideline recommends a threshold of systolic blood pressure (SBP) equal to or greater than 135 mm Hg and/or diastolic blood pressure (DBP) equal to or greater than 85 mm Hg for automated office blood pressure measurement (OBPM) with at least three readings take during the same visit, discarding the first reading and averaging the latter two (or >  = 140/90 mm Hg for manual office blood pressure measurement) for the diagnosis of hypertension [5]. If a patient meets these blood pressure thresholds with OBPM, then ambulatory (ABPM) or home (HBPM) blood pressure measurements are recommended to rule out white coat hypertension (individuals who are hypertensive when measured in office but normotensive in other settings [6]), with thresholds of 135/85 mm Hg used for diagnosis (or >  = 130/80 for 24-h mean for ABPM). Their guidelines differ for individuals with diabetes, where a threshold of manual OBPM >  = 130/80 for 3 or more measurements on different days is recommended for hypertension diagnosis [5].

European and UK standards for the diagnosis of hypertension are similar, with an office-based measurement threshold of > 140/90 followed by confirmatory measurements [7, 8]. In the USA, the American College of Cardiology (ACC) and American Heart Association (AHA) 2017 define hypertension thresholds by stage (stage 1: SBP 130–139 mm Hg and/or DBP 80–89 mm Hg; stage 2: ≥ 140 mm Hg and/or ≥ 90 mm Hg) measured by at least two high-quality measurements obtained on two or more separate occasions [9].

### Description of disease burden

Hypertension is ranked as the leading risk factor for cardiovascular morbidity and death globally [10, 11]. Hypertension is also recognized as the number one contributor to disability-adjusted life years, a measure of overall disease burden defined as the number of years lost due to poor health, disability, or death [10] and is the most common reason for primary care visits in developed countries [12]. The global age-standardized prevalence of hypertension in adults in 2010 (defined as a blood pressure greater than or equal to 140/90 mm Hg) was 31.1% in high-income countries and 31.5% in low- and middle-income countries [11, 13]. A review of population-based Canadian surveys found that while the prevalence of hypertension had remained stable between 1992 and 2009, the rates of controlled hypertension (participants with previously diagnosed hypertension with a blood pressure of < 140/90 mm Hg) had increased, reflecting increases in awareness and treatment [14]. This trend may be shifting, as more recent Canadian data from 2007 to 2017 showed deterioration in hypertension awareness, treatment, and control, especially for older women [15, 16]. Additionally, deterioration in blood pressure control may have been further exacerbated by the COVID-19 pandemic [17]. A recent UK report estimated that almost half a million individuals missed out on treatment of high blood pressure due to COVID-19 [18]. The 2016–2019 Canadian Health Measures Survey revealed a hypertension prevalence of 22.6% (defined as an average blood pressure measurement of >  = 140/90 mm Hg over five readings or self-reported use of antihypertensive medications) in Canadians aged 20–79 years and an increase from 19.6% of adults reported in 2007–2009 [19]. However, this is not age adjusted and may be reflective of the aging Canadian population.

Healthcare organizations and professionals have made substantial efforts to reduce the burden of hypertension by increasing hypertension awareness, treatment, and control [20]. One study found that 84% of Canadians aged 20 to 79 with hypertension were aware of their condition between 2012 and 2015. However, young Canadians aged 20 to 39 were much less likely to be aware of being hypertensive (65%) than older individuals [21].

### Risk factors

Blood pressure is regulated by a complex system of neurohumoral factors; an imbalance in any of these factors could contribute to the development of hypertension [22]. Hypertension that is caused by other conditions, such as primary hyperaldosteronism, renal disease, or obstructive sleep apnea, is referred to as secondary hypertension [23]. Most patients (90–95%) have primary or “essential” hypertension, in which no cause has been identified [22, 23]. The pathophysiological mechanisms of primary hypertension are thought to be multifactorial, involving both lifestyle and genomic factors [22, 24]. Non-modifiable risk factors include increasing age [25, 26], family history of hypertension [25, 27], and other comorbidities, such as type 2 diabetes mellitus or chronic kidney disease [5]. Modifiable lifestyle risk factors associated with increased risk of hypertension include excessive salt intake [28–30], low intake of fruits and vegetables [31–34], physical inactivity [32, 35, 36], alcohol consumption [32, 37, 38], tobacco smoking [27, 39], and being overweight or obese [25, 27, 32, 40, 41]. In North America, the prevalence of hypertension is higher in Black individuals compared with white individuals, as well as in individuals with South Asian or Indigenous ancestry [42]. These differences in risk may be largely explained by dietary patterns, smoking, and social factors such as socioeconomic status [42–45] in addition to other contributors [46, 47].

### Consequences of hypertension

Cardiovascular consequences include increased risk of angina, myocardial infarction, congestive heart failure, peripheral arterial disease, and stroke [3]. Beyond cardiovascular disease, hypertension is also a major risk factor for chronic kidney disease [48, 49], dementia [50, 51], retinopathy [52], and encephalopathy [53]. Hypertension is a leading modifiable risk factor for cardiovascular morbidity and mortality and all-cause mortality globally, and in Canada [54, 55], high blood pressure is estimated to contribute to more than 10% of the population-attributable fraction of premature deaths worldwide [56]. Globally, high blood pressure is associated with 15.2% of all deaths and 7.4% of all premature death or disability, and there have been numerous calls to action to diagnose and control hypertension to prevent negative health effects [15, 57–60]. A systematic review evaluated the risk of cardiovascular events and found those with high normal blood pressure (130–139 and 85–89 mm Hg) had an increased risk of cardiovascular events (risk difference 0.69, 95% *CI* 0.43 to 0.97 per 1000 person years) compared to individuals with low normal or low blood pressure [61]. Associations were also seen for those with grade 1 hypertension (1.81, 95% *CI* 1.34 to 2.34 per 1000 person years) and grade 2 hypertension (4.24, 95% *CI* 2.58 to 6.48 per 1000 person years).

### Screening for hypertension

Screening aims to detect high blood pressure in people who are asymptomatic and who do not have a previous diagnosis of hypertension. As hypertension rarely has early symptoms prior to an adverse outcome, it is most often not identified without screening [62]. In a 2017 survey of Canadian family physicians, the majority of physicians reported that manual OBPM with a mercury or aneroid device and stethoscope was their most frequent method to screen patients for hypertension, with automated OBPM being the second most popular screening method [63]. OBPM is subject to sources of error, including the white coat phenomenon [6, 64] and errors in the measurement procedure by the blood pressure taker [65–67]. Blood pressure measurement through ABPM and HBPM methods is therefore recognized as superior to OBPM in accuracy [68] and more strongly associated with cardiovascular morbidity and mortality [69–71]. However, there is emerging evidence that unattended (no medical personnel in the room) and fully automated OBPM assessment is comparable to awake ambulatory BP readings and may therefore minimize the “white coat” effect [68]. The American College of Cardiology (ACC) and American Heart Association (AHA) 2017 guidelines recommend OBPM both as a screening method for hypertension and to confirm the diagnosis [9]. Standard screening includes routine blood pressure measurements at appropriate clinic visits, regardless of previous measures or the interval since the last measure. Although this approach is simple, it has been suggested that a more nuanced strategy around screening intervals, such as risk-based screening intervals, may be more efficient for the prevention of cardiovascular disease [72–74]. Practitioners would benefit from clearly defined optimal screening methods, frequency, and target population.

Given the risk of cardiovascular disease, hypertension screening could provide a benefit if previously unrecognized hypertension is diagnosed and brought under control. Evidence supports the efficacy of treating hypertension, both through pharmacological therapies [75–78] and lifestyle interventions [29, 79–81]. However, screening programs for hypertension can harm persons, for example, through labeling, overdiagnosis, or overtreatment [82–84]. Hypertension requires lifelong management, and potential harms, such as psychological effects, adverse effects from medications, and increased burden on both the individual themselves and the healthcare system, must be weighed against the benefits of screening.

### Evidence-based recommendations

In 2012, the Canadian Task Force on Preventive Health Care (“Task Force”) published recommendations on screening for hypertension in adults. Based on moderate-quality evidence from their systematic review, the Task Force recommended the following: (1) blood pressure measurement at all appropriate primary care visits (“appropriate” visits may include periodic health visits, urgent office visits for neurologic or cardiovascular-related issues, medication renewal visits, and other visits where the primary care practitioner deems it appropriate), (2) that blood pressure be measured according to the current techniques described in the 2012 Canadian Hypertension Education Program (CHEP) recommendations for office and out-of-office blood pressure measurement (see Additional file 1) [85], and (3) for people with elevated blood pressure measurement during screening, the 2012 CHEP criteria for assessment and diagnosis of hypertension should be applied to determine whether patients meet diagnostic criteria for hypertension [86, 87]. In 2015, the US Preventive Services Task Force (USPSTF) recommended screening for high blood pressure in adults aged 18 years or older and obtaining measurements outside of the clinical setting for diagnostic confirmation before starting treatment [88]. Regarding screening intervals, the USPSTF recommended annual screening for adults aged 40 years or older and those at increased risk for high blood pressure (i.e., high-normal blood pressure [130 to 139/85 to 89 mm Hg], overweight or obese, and African American). They suggest adults aged 18 to 39 years with normal blood pressure (i.e., < 130/85 mm Hg) and without risk factors be rescreened every 3 to 5 years. The USPSTF released an updated evidence review [89] and hypertension screening recommendations in April 2021 and reaffirmed their 2015 recommendations [90]. Hypertension Canada released guidelines for prevention, diagnosis, risk assessment, and treatment of hypertension in adults and children in 2020. They recommended that healthcare professionals trained to measure blood pressure should assess blood pressure in adults at all appropriate visits to determine cardiovascular risk and monitor antihypertensive treatment [5]. Regarding antihypertensive treatment initiation, Hypertension Canada promotes a risk-based approach to treatment thresholds, with low-risk patient populations (no target organ damage or CVD risk factors) having a threshold of SBP >  = 160 mm Hg and/or DBP >  = 100 mm Hg. The treatment initiation BP threshold is lower (SBP ≥ 130) for those at high risk of CVD (e.g., chronic kidney disease, Framingham risk score >  = 15%, age >  = 75 years) or those with diabetes mellitus (SBP ≥ 130 and/or DBP ≥ 80) [5].

### Rationale, key questions, and approach

**Fig. 1:**
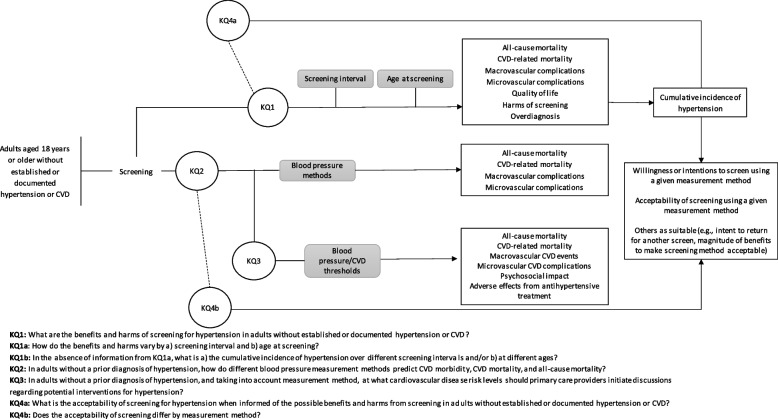
Analytic framework

The Task Force is updating their 2012 guideline on hypertension screening in adults because new recommendations and relevant systematic reviews have been published since the original Task Force guideline. Further, the Task Force methods have evolved since 2012 and now consider evidence on patient values and preferences for screening and of screening methods. The hypertension working group will use the evidence from the planned systematic reviews to develop updated recommendations for primary care providers on hypertension screening. The key questions to be addressed are available in Table 1. Figure 1 presents the analytic framework of the KQs, relevant population, interventions, and outcomes to be considered.

**Table 1:** Key questions to inform an update of recommendations by the task force on hypertension screening in adults aged 18 years and older in primary care

**Key questions**	
**KQ1**	What are the benefits and harms of screening for hypertension in adults?
**KQ1a**	How do the benefits and harms vary by (a) screening interval and (b) age at screening?
**KQ1b**	What is the cumulative incidence of hypertension (a) over different screening intervals and/or (b) at different ages?
**KQ2**	In adults without a prior diagnosis of hypertension, how do different blood pressure measurement methods predict CVD morbidity, CVD mortality, and all-cause mortality?
**KQ3**	In adults without a prior diagnosis of hypertension, and taking into account measurement method, at what cardiovascular disease risk levels should primary care providers initiate discussions regarding potential interventions for hypertension? This guideline question will be addressed in this review by answering the key question: “What is the effectiveness of initiating antihypertensive drug treatment at differing blood pressure thresholds or cardiovascular disease risk levels?”
**KQ4a**	What is the acceptability of screening for hypertension when informed of the possible benefits and harms from screening in adults?
**KQ4b**	Does the acceptability of screening differ by measurement method?

## Methods

### Protocol development

This protocol was developed by the Evidence Review and Synthesis Centre (ERSC) at the University of Ottawa (A. B. 1, A. B.2, N. S., B. S., D. M., M. B., J. L., J. F., J. K., F.L., K.P.) in consultation with the hypertension working group consisting of Task Force members (B. J. W., R. G., N. P., G. T. 1, B. D.T.), and with support from working group external clinical experts (C. E. C., J. K., P. L.), and the Science Team (C. G., M. S., G. T. 2). The full Task Force has approved this final version of the protocol, and peer reviewers and stakeholders have reviewed it. The methodology planned for the systematic reviews will follow the Task Force methods manual [91] with additional guidance from the Cochrane Handbook [92] and GRADE handbook [93].

Reporting of this protocol was completed using the Preferred Reporting Items for Systematic Reviews and Meta-Analyses Protocols (PRISMA-P) checklist [94] (see Additional file 2). The protocol will be registered on PROSPERO. In addition, the protocol will be available on the Open Science Framework (osf.io/8w4tz). The working group, external clinical experts, and Science Team will not be involved in selecting studies, data extraction, or data analysis but may be consulted for advice if required. The ERSC will make all final decisions, and any amendments to the reviews and this protocol will be provided in the final manuscript.

Following development of an extensive scoping and refinement exercise led by the Science Team, the hypertension working group established and finalized the key questions and related PICOTS (population, interventions, comparators, outcomes, timing, and setting) with involvement from the entire Task Force, the ERSC, and the Science Team.

For KQ1, the working group considered available systematic reviews and decided to use the recent 2021 USPSTF review and their relevant key questions (KQ1 and KQ4) on the benefits and harms of hypertension screening as it aligns with the working group’s desired criteria and was judged to be of high quality using the AMSTAR-2 tool (Additional file 3) [89]. These 2021 USPSTF key questions will also be used to examine evidence on how benefits and harms vary by screening interval or age at screening (KQ1a) or, in the absence of data, what is the cumulative incidence of hypertension over different screening intervals and/or at different ages (KQ1b). The ERSC will not undertake updated searches of the USPSTF review. This topic does not have a rapidly evolving evidence base. To our knowledge, there have not been any screening trials published since the 2012 guideline that we would expect to change screening recommendations. Any additional new harms related to HBPM will be examined through targeted searches at the time of guideline development and will be addressed narratively. De novo systematic reviews will be conducted to address KQ2 and KQ4.

An overview of reviews will be undertaken to address KQ3. An overview approach was selected to maximize review efficiency, as there is a large evidence base of primary studies addressing treatment initiation for hypertension, as well as several high-quality systematic reviews that have summarized these primary studies. An overview approach will also enable us to explore concordance/discordance between existing systematic reviews in this area, where conflicting review results have previously been reported [95]. The methodology planned for the overview of reviews will be informed by the Cochrane Handbook (Chapter 5) [96], with additional supplementary guidance on overview methodology [97–99]. To maximize efficiency and avoid duplication of efforts, we will use the National Institute for Health and Care Excellence (NICE, UK) 2019 review for initiating treatment of hypertension as the basis for our overview [100]. The KQ1 of the NICE review aligns with the working group’s desired criteria for KQ3, and the review captured systematic reviews of treatment initiation published since 2000. We will examine systematic reviews that were captured in the 2019 UK NICE review for inclusion (see the “Study selection” for further details on review selection) and search for any new systematic reviews that have been published since its conduct.

For KQ2 and KQ3, members of the working group developed a list of preliminary outcomes for key questions KQ2 and KQ3. For KQ1, outcomes were limited to those included in the 2021 USPSTF systematic review [89]. Through consensus, the outcomes for KQ1–KQ3 were rated by six working group members according to GRADE methodology as *critical* (rated 7 to 9 out of 9), *important* (rated 4 to 6 out of 9), or of *limited importance* (rated 1 to 3 out of 9) for making guideline recommendations [101]; only *critical* and *important* outcomes were retained for the systematic reviews. Outcomes related to KQ4 (acceptability) underwent a separate rating process.

The working group initially rated 11 outcomes as *critical* or *important*. Through consensus, it was decided that individual CVD-related morbidity outcomes would be collapsed into two categories: macrovascular CVD events (e.g., myocardial infarction, stroke, peripheral arterial disease) and microvascular complications (e.g., renal disease, retinal disease), thus collapsing into two versus five outcomes. Further, ‘overtreatment,’ although originally rated as an *important* outcome, was excluded given adverse effects of antihypertensive treatment, and overdiagnosis is already included. Therefore, a total of seven outcomes were included (see Table 2).

**Table 2:** Final set of outcomes deemed to be of critical or important for guideline development and decision-making

Outcomes	Priority
***Potential benefit of reduced***	
All-cause mortality	Critical
CVD-related mortality	Critical
Macrovascular complications (e.g., myocardial infarction, stroke, peripheral arterial disease)	Critical
Microvascular complications (e.g., renal disease, retinal disease)	Important
***Potential harm of increased***	
Adverse effects of antihypertensive treatment	Important
Overdiagnosis ^a^	Important
Psychosocial impact of screening	Important

^a^The issue of overdiagnosis in hypertension is complex. Hypertension may be considered either a disease or a risk factor for cardiovascular events. We may dichotomize individuals as being hypertensive or not or assign them a risk of future event. These distinctions and the different recommended thresholds for diagnosis are important considerations in estimating the magnitude of overdiagnosis in hypertension

### Eligibility criteria

The inclusion and exclusion criteria for KQ1, KQ2, KQ3, and KQ4 are listed in Tables 3, 4, 5, and 6. The working group will rely on the 2021 USPSTF systematic review and their KQ1 and KQ4 on the benefits and harms of hypertension screening [89].

**Table 3:** Key question 1, 1A, 1B eligibility criteria, from USPSTF 2021 review (KQ1: What are the benefits and harms of screening for hypertension in adults? KQ1a: How do the benefits and harms vary by (a) screening interval and (b) age at screening? KQ1b: What is the cumulative incidence of hypertension (a) over different screening intervals and/or (b) at different ages?)

	**Inclusion criteria**	**Exclusion criteria**
**Aim**	Screening for hypertension in a primary care setting	Studies measuring blood pressure for reasons other than screening or confirmation of a hypertension diagnosis; mathematical transformation of blood pressure results (e.g., pulse pressure, variability) or diurnal variations (e.g., morning surge, dipping) for use as additional diagnostic criteria, predicting risk, or both
**Population**	Adults age ≥ 18 years	Pregnant women, children (age < 18 years), inpatients, persons in institutions, patients with secondary hypertension, and highly selected groups of patients (e.g., those with chronic kidney disease or renal transplant) who do not represent a primary screening population Patients treated for hypertension with medication
**Interventions**	*Benefits & harms*: Clinic-based, noninvasive brachial blood pressure measurement (manual or attended/unattended automated) using any common device or screening protocol during a single encounter *Harms*: HBPM and ABPM	*Benefits & harms*: Blood pressure measurement with wrist and finger monitors, forearm cuffs, or ankle and toe measures; any method not commonly used in routine blood pressure screening (e.g., invasive methods, noninvasive method of central blood pressure measurement); Osler’s maneuver *Benefits*: HBPM and ABPM
**Comparator**	No blood pressure measurement with the intention of screening	
**Outcomes**	Potential benefits of the following: 1. Reduced all-cause mortality 2. Reduced CVD-related mortality 3. Reduced macrovascular CVD events (cardiovascular disease events, including myocardial infarction, sudden cardiac death, stroke, heart failure, and hospitalization for coronary heart disease, symptomatic peripheral arterial disease) 4. Reduced microvascular CVD events (end-stage renal disease, vascular dementia) Potential harms: 5. Increased harms of screening (e.g., labeling, absenteeism, quality of life measures, tolerability of ABPM devices) 6. Increased overdiagnosis^a^ Potential benefits or harms: 7. Increased/decreased quality of life	Cardiovascular symptoms (e.g., palpitations), angina pectoris (chest pain), revascularization, carotid intima-media thickness, left ventricular hypertrophy, or patient satisfaction
**Timing of outcome assessment**	No restrictions	No restrictions
**Setting**	Eligible primary care settings must have physicians or personnel trained in blood pressure measurement, established blood pressure measurement protocols, and ongoing documentation procedures	Settings not generalizable to primary care, inpatient/residential facilities
**Study design**	*Benefits*: Randomized controlled trials (RCTs) CTs and controlled clinical trials (CCTs) *Harms*: RCTs, CCTs, and cohort studies	*Benefits & harms*: Before-after studies, time series, case series, case reports, case–control studies, and simulation studies *Harms*: Cross-sectional studies
**Country**	Studies conducted in countries categorized as “very high” on the 2015 Human Development Index (as defined by the United Nations Development Programme)	Studies conducted in countries not categorized as “very high” on the 2015 Human Development Index
**Language**	English^b^	N/A
**Study quality**	Fair or good quality^b^	N/A

^a^We will review included/excluded studies from the 2021 USPSTF systematic review to capture any information on overdiagnosis, as this was not an outcome originally included in the 2021 USPSTF review. Overdiagnosis will be addressed as part of the analysis at the synthesis stage. Outcome data for overdiagnosis will be extracted as reported by study authors

^b^The USPSTF 2021 systematic review excluded studies published in languages other than English and studies deemed to be of poor quality (i.e., fatally flawed). We will review studies excluded for these reasons at the full-text stage and include these studies if they meet our other eligibility criteria for KQ1. Citation: Guirguis-Blake JM, Evans CV, Webber EM, Coppola EL, Perdue LA, Weyrich MS (2021) Screening for Hypertension in Adults: An Updated Systematic Evidence Review for the U.S. Preventive Services Task Force. https://www.uspreventiveservicestaskforce.org/uspstf/document/final-evidence-review/hypertension-in-adults-screening

**Table 4:** Key question 2 eligibility criteria (In adults without a prior diagnosis of hypertension, how accurately do different blood pressure measurement methods predict CVD morbidity, CVD mortality, and all-cause mortality?)

	**Inclusion**	**Exclusion**
**Population**	Adults aged 18 years or older without established or documented hypertension or CVD A staged approach will be used to potentially consider indirect evidence for our population. We will consider populations of adults on antihypertensive medication or with documented hypertension, if we fail to find evidence on adults without documented hypertension or CVD	Pregnant women, children (age < 18 years), inpatients, persons in institutions, patients with secondary hypertension, and highly selected groups of patients (e.g., those with chronic kidney disease or renal transplant) who do not represent a primary screening population
**Interventions**	Blood pressure measured using any clinic-based noninvasive brachial measurement including manual OBPM and attended or unattended automated OBPM. Home or ambulatory blood pressure measurement with any measurement protocol	Non-brachial measures (e.g., blood pressure measurement with wrist and finger monitors, forearm cuffs, or ankle and toe measures), instruments requiring specialist expertise, personal wearable smartphone “apps”/devices, or similar
**Comparator**	Blood pressure measured using any other noninvasive brachial clinic-based, home, or ambulatory blood pressure measurement (with any measurement protocol)	Non-brachial measures (e.g., blood pressure measurement with wrist and finger monitors, forearm cuffs, or ankle and toe measures), instruments requiring specialist expertise, personal wearable smartphone “apps”/devices, or similar
**Outcomes**	Measures of association (e.g., risk ratios, hazard ratios) between BP levels measured at baseline using eligible measurement methods: 1. All-cause mortality 2. CVD-related mortality 3. Macrovascular CVD events (e.g., stroke, myocardial infarction) 4. Microvascular CVD complications (e.g., renal disease, retinal disease)	N/A
**Study design**	Eligible studies include comparative studies that follow a cohort of subjects over time and report the association of different BP measurement methods at baseline with outcomes of interest over follow-up Eligible designs include RCTs, prospective or retrospective cohort studies, nested case–control studies, within-arm analyses of intervention studies	Non-nested case–control studies, before-after studies, time series, case series, simulation studies, editorials, commentaries
**Language**	English and French	Any other language
**Setting**	Primary care and community-based settings (e.g., pharmacy) No country-based restrictions	Inpatient or medical specialist settings (e.g., hospital, ICU, specialist’s office)
**Publication date**	No limitation	N/A
**Study quality**	No restrictions	N/A

**Table 5:** Key question 3 eligibility criteria (In adults without a prior diagnosis of hypertension, and taking into account measurement method, at what cardiovascular disease risk levels should primary care providers initiate discussions regarding potential interventions for hypertension?)

	**Inclusion**	**Exclusion**
**Population**	Reviews of adults aged 18 years or older who are not on current pharmacological treatment for hypertension	Reviews exclusively in individuals < 18 years, pregnant women Reviews of patients with secondary hypertension and highly selected groups of patients (e.g., those with chronic kidney disease or renal transplant)
**Interventions**	Treatment initiation at a lower threshold^a^ • Systolic blood pressure targets: 110–119 mmHg, 120–129 mmHg, 130–139 mmHg, 140–59 mmHg, 160 mmHg, or above • Diastolic blood pressure targets: 75–79 mmHg, 80–84 mmHg, 85–89 mmHg, 90–94 mmHg, 95 mmHg, or above • Cardiovascular risk thresholds: (1) 5–9%, (2) 10–14%, (3) 15–19%, (4) above 20%	N/A
**Comparator**	Treatment initiation at higher blood pressure and/or cardiovascular risk thresholds	• Noncomparative data where all participants start at the same treatment threshold • Studies do not stratify by two or more baseline blood pressure or CVD risk groups
**Outcomes**	Potential benefits 1. Reduced all-cause mortality 2. Reduced CVD-related mortality 3. Reduced macrovascular CVD events (e.g., stroke, myocardial infarction) 4. Reduced microvascular CVD complications (e.g., renal disease, retinal disease) Potential harms 1. Increased psychosocial impact (e.g., stress) 2. Increased adverse effects from antihypertensive treatment	N/A
**Study design**	Systematic reviews of randomized controlled trials (RCTs)^b,c^	Primary studies, editorials, commentaries
**Language**	English and French	Any other language
**Setting**	Reviews in primary care and community-based settings (e.g., pharmacy) No country-based restrictions (for systematic reviews or included primary studies)	Reviews in inpatient or medical specialist settings (e.g., hospital, ICU, specialist’s office)
**Publication date**	2018-present	N/A
**Study quality**	No restrictions	N/A

^a^The BP measurement method will be recorded, and data will be presented by both BP/CVD risk threshold and measurement method, when available. Intervention treatment categories may be recategorized depending on what is reported in systematic reviews and our findings in KQ2.

^b^Reviews will be considered systematic if they meet the four following criteria: (1) searches at least one database, (2) reports their selection criteria, (3) conducts quality or risk-of-bias assessment on included studies, and (4) provides a list and synthesis of included studies.

^c^Systematic reviews that include non-randomized studies will also be included if they report results from RCTs separately

**Table 6:** Key question 4a and 4b eligibility criteria (KQ4a: What is the acceptability of screening for hypertension when informed of the possible benefits and harms from screening in adults? KQ4b: Does the acceptability of screening differ by measurement method?)

	**Inclusion**	**Exclusion**
**Population**	Adults aged 18 years or older without established or documented hypertension or CVD	Individuals < 18 years. Adults with established or documented hypertension or CVD
**Interventions**	Participants are provided with information on the relative magnitude of benefits and harms of screening for hypertension using any clinic-based, home, or ambulatory blood pressure measurement. An alternative is when investigators solicit the magnitude of benefits and/or harms where screening is acceptable **KQ3b**: Subgroup analyses of acceptability by screening method (e.g., clinic, home, ambulatory measurement methods)	N/A
**Comparator**	Depending on the study design, comparator may be no screening, another form of screening, or a different form of information that does not include the magnitude of effects for benefits and harms	N/A
**Outcomes**	Acceptability measures • Willingness or intentions to screen using a given measurement method • Acceptability of screening using a given measurement method • Others as suitable (e.g., intent to return for another screen, magnitude of benefits to make screening method acceptable)	N/A
**Study design**	RCTs, CCTs, observational studies with control groups that assess patient acceptability of screening	Systematic reviews, cost-effectiveness studies, qualitative studies, case report, and case series Analyses of data that were not reported by patients (e.g., databases of health records) or on outcomes outside the perspective of individuals considering screening for hypertension Studies reporting only access to screening and studies on knowledge or awareness about screening. Studies reporting only outcome prioritization, time trade-off, health state values, or willingness to pay
**Language**	English and French	Any other language
**Setting**	Any setting, no country-based restrictions	N/A
**Publication date**	No limitation	N/A
**Study quality**	No restrictions	N/A

### Information sources and search strategy

Draft search strategies (Additional file 4) have been developed by an experienced medical information specialist and tested through an iterative process in consultation with the review team. Prior to running the final searches, the MEDLINE strategies for each KQ will be peer reviewed by another senior information specialist using the PRESS checklist [102] (see Additional file 5). With the exception of the additional database, Epistemonikos, searched for KQ3, all databases will be searched on the Ovid platform in multifile mode, using the Ovid deduplication feature before downloading the results. Results will be downloaded and deduplicated using EndNote (Clarivate Analytics) and uploaded to DistillerSR.

- *KQ1*: No new searches will be conducted for KQ1, as we are relying on the USPSTF 2021 review.
- *KQ2*: For KQ2, we will search Ovid MEDLINE® ALL, Embase Classic + Embase, APA PsycInfo, and EBM Reviews—Cochrane Central Register of Controlled Trials (CENTRAL) with no date limits. Draft strategies utilize a combination of controlled vocabulary (e.g., “blood pressure,” “cardiovascular diseases,” “risk assessment”), and keywords (e.g., “sphygmomanometer,” “cardiac disease,” “risk factor”). Vocabulary and syntax will be adjusted across the databases, and filters for RCTs, cohort studies, and other designs of interest will be applied in all databases except CENTRAL. No date limits will be applied.
- *KQ3*: For KQ3, we will search Ovid MEDLINE® ALL, Embase Classic + Embase, and APA PsycInfo, as well as Epistemonikos. The draft strategies utilize a combination of controlled vocabulary (e.g., “hypertension,” “antihypertensive agents,” “heart disease risk factors”), and keywords (e.g., “high blood pressure,” “diuretic,” “risk factor”), with vocabulary and syntax adjusted across the databases. A filter for systematic reviews and meta-analyses will be applied. As the 2019 UK NICE review searched for systematic reviews prior to 2018, we will search from 2018 until present.
- *KQ4*: For KQ4, we will search Ovid MEDLINE® ALL, Embase Classic + , and APA PsycInfo (no date limits). The draft strategies utilize a combination of controlled vocabulary (e.g., “hypertension,” “mass screening,” “patients/px [psychology]”), and keywords (e.g., “high blood pressure,” “early recognition,” “trade-off”). Vocabulary and syntax will also be adjusted across the databases. We applied filters for RCTs, controlled clinical trials, and observational studies. For KQ2, KQ3, and KQ4, animal-only records, opinion pieces, and conference abstracts will be removed where possible, and results will be limited to English or French.

We will supplement the electronic database search strategies with gray literature sources (i.e., sources other than peer-reviewed journals). We will follow the Canadian Agency for Drugs and Technologies in Health (CADTH) Grey Matters checklist [103] for relevant gray literature sources. The CADTH checklist includes health technology assessment agencies, guideline organizations, clinical trials registries, search engines, and additional databases. In addition to the CADTH checklist, we will search websites of relevant organizations as suggested by the working group and clinical experts. The full list of websites is available in Additional file 6.

Preprints will be eligible for inclusion in our de novo systematic reviews (KQ2/KQ4) and overview of reviews (KQ3) and handled based on the methodological considerations for use of preprints in evidence syntheses by Clyne and colleagues [104]. We will review bibliographic databases policies and coverage to ensure capture of relevant preprints. If preprints are included, we will check peer review status pre-specified intervals (full-text retrieval stage, results synthesis, search updates). If a final peer-reviewed version is found, we will check for differences between the preprint and the peer-reviewed version, and sensitivity analyses will be performed to assess the impact of inclusion of preprints on the overall review results and conclusions.

### Study selection

Search results will be downloaded and deduplicated using EndNote (Clarivate Analytics) [105]. Results will be uploaded into the DistillerSR (Evidence Partners, Ottawa, Canada) online screening and extraction platform [106]. Screening forms for title and abstract screening and full-text review will be developed and pilot tested on a random sample of 50 titles and abstracts and 25 full-text articles or five reviews for KQ3. Any disagreements among reviewers will be resolved by discussion, and adjustments to the form will be completed as required. Pilot testing will continue until the disagreement rate between reviewers is low (i.e., < 5%).

Title and abstract screening will be completed independently by reviewers using the liberal accelerated approach [107]. This approach allows records that one reviewer selects as either potentially relevant (i.e., included) or unclear about relevance to advance to full-text review without a second reviewer. Any record labelled as excluded will be screened by two reviewers to confirm the decision to exclude. Resolution about disagreements will not be required during this stage. Full-text review will be completed independently and in duplicate by reviewers. Any discrepancies will be resolved by consensus among the reviewers or by a third reviewer.

If articles are not available electronically, we will request access through the university library interlibrary loan service. Further, we will contact the corresponding author (by email with a maximum of three attempts) for published or unpublished reports or data. Similarly, we will search to see if a corresponding publication exists for protocols of potentially relevant studies that we identify. Otherwise, we will contact the corresponding author to determine the publication status. We will review the included studies of related evidence-based guidelines and knowledge syntheses that were identified as part of the scoping and refinement exercise and from the electronic database and gray literature searches.

If an article lacks sufficient information for us to decide on eligibility, we will contact the corresponding author for additional information (by email with a maximum of three attempts). If a response is not received, we will exclude the article. We may consult with the working group and clinical experts for advice on potentially eligible studies. When consulting with the working group, we will anonymize the article to avoid study and data identification. The decision on eligibility will be determined independently by the ERSC. For the excluded studies, we will provide a list of excluded studies with reasons for exclusion, and the study selection process will be documented in a PRISMA flow diagram [108].

#### KQ1

For KQ1, a systematic review will not be conducted, and the working group will rely on the results for the relevant KQs in the 2021 USPSTF systematic review. However, we will review the 2021 USPSTF systematic review and their included and excluded studies to confirm that they meet the working group criteria and Task Force procedures (e.g., including French language publications and handling of studies deemed as of “poor quality”) [89]. We will also review included/excluded studies from the 2021 USPSTF systematic review to capture any information on overdiagnosis, as this was not an outcome originally included in the 2021 USPSTF review.

#### KQ3

For our overview of reviews (KQ3), study selection will also be informed by a process of data mapping, as there is a high likelihood that we will detect multiple systematic reviews that address the same research question (i.e., PICO criteria). These reviews will likely rely on the same evidence base, resulting in “overlap” (multiple systematic reviews that include the same primary studies) [96]. To address overlap, once eligible systematic reviews have been identified, we will map their research questions (i.e., PICO criteria) and review characteristics (i.e., search dates, comprehensiveness, and quality, as determined by AMSTAR-2). When multiple systematic reviews address the same research question, we will compare review characteristics. Reviews will be excluded if a more recent review of similar (or higher) methodological quality has been detected and if they contain no additional primary studies of interest or analyses to a more recent review [97]. In the cases of overlap where reviews cannot be excluded, we will calculate the degree of primary study overlap across systematic reviews using the corrected covered area (CCA) [109]. CCA will be calculated according to the protocol described in Pieper et al., with CCA of 0–5% representing slight overlap, 6–10% moderate overlap, 11–15% high overlap, and > 15% very high overlap [109]. We will calculate CCA at the outcome level, as well as pairwise CCA (the degree of overlap for an outcome between two reviews). A citation matrix will also be presented for each outcome to visualize the degree of overlap [109].

We will perform this process for both the systematic reviews captured in the 2019 UK NICE review, as well as any new systematic reviews found in our search update. Mapping of review characteristics will be performed by a single reviewer with verification by a second reviewer. The decision to exclude a review will be based on the aforementioned criteria, through consensus by at least two reviewers, and with additional review by the hypertension working group. When overlapping systematic reviews are included in the overview, the level of agreement between review results will be explored (see “Synthesis of included studies” section).

### Data extraction

We will develop standardized extraction forms in DistillerSR and pilot test the forms on a random sample of five included studies for each KQ [106]. Any data extraction differences among the reviewers will be resolved by discussion or consulting with a senior reviewer. Adjustments to the forms will be completed as appropriate. Data extraction will be completed independently and in duplicate by reviewers. Any discrepancies will be resolved by consensus among the reviewers or by a senior reviewer. The preliminary data extraction items for each KQ are available in Additional file 7. Data will be reformatted and presented in the text and tables of the final manuscript as needed. If information is missing or unclear, then we will contact the corresponding author of the study for the required information thrice by email over 1 month. For multiple publications of the same study, we will extract data from the most recent publication, and the previous publications will be used as secondary sources.

#### KQ3

For our overview of reviews (KQ3), all relevant data (Table 5) will be extracted as they were synthesized/reported in the included systematic reviews. We will also extract risk-of-bias assessments directly from the included systematic reviews. We will not consult primary studies for additional information or verification of the data reported in the systematic review. If systematic reviews report a meta-analysis for an outcome, we will collect the pooled effect estimates with their associated confidence intervals and heterogeneity tests. For reviews that do not conduct a meta-analysis, we will extract outcome data based on the reporting in the review. In the case of no optimal quantitative data, we will extract a narrative summary of findings from the reviews.

If we identify discrepant data reported from primary studies in overlapping systematic reviews, we will review both systematic reviews to attempt to identify the source of the discrepancy. If we are unable to reconcile the discrepancies, we will contact the review authors to verify the information. Similarly, if risk-of-bias assessments in the systematic reviews are flawed, incomplete, or missing, we will attempt to contact the primary study author to verify the information. If we are unable to obtain complete risk of bias assessments, we will perform new risk of bias assessments using the methods outlined in the “Risk-of-bias assessment” section for primary studies.

In the case that a systematic review is partially in scope and only some of the included primary studies meet the eligibility criteria (e.g., inclusion of trials conducted in adolescents), we will determine if the review analyses are sufficiently direct to inform our key question. We will examine the relative contribution of the primary studies to the analysis presented in the systematic review synthesis. If results/analyses in the review are stratified by this factor, we may only include data that meet our eligibility criteria (e.g., include review results for adults only). Final inclusion or exclusion will be reviewed by the working group for their input, and all decisions will be documented and transparently reported in the final overview report.

### Risk-of-bias assessment

Forms for the risk-of-bias assessments will be developed in DistillerSR [106]. Reviewers will pilot test each study design form for a random sample of five included studies. Any conflicts among reviewers will be resolved by discussion or by a third reviewer. Assessments will be completed independently and in duplicate by reviewers using the appropriate study-specific tool for the design of the included study. Any disagreements in the assessments will be resolved by consensus among the reviewers or by a senior reviewer.

#### KQ2/KQ4

We will use study design-specific tools that best account for potential sources of bias. For randomized and non-randomized controlled trials (KQ2, KQ4), we will use the Cochrane risk-of-bias tool for randomized controlled trials (version 2.0) [110], as recommended by the Task Force methods manual [111]. The outcome-specific domains (e.g., blinding of outcome assessors) will be assessed for each outcome within the study deemed to be of critical or important consequence (see Table 2) [112]. We will use the Agency for Healthcare Research and Quality guidance on assessing outcome and analysis reporting bias [113]. For cluster randomized trials, we will assess recruitment bias (when participants are recruited after the randomization of clusters) in the “other sources of bias” domain of the Cochrane tool [114]. We will rate the overall risk of bias as “low” if all the domains are low risk, “high” if at least one domain is high risk, or “unclear” if at least one domain is unclear, and no other domains are high risk. For observational studies (cohort and case control) (KQ2, KQ4), we will use the Newcastle–Ottawa scale [115], and the QUIPS (Quality In Prognosis Studies) tool will be used for predictor finding studies (KQ2) [116].

#### KQ3

For our overview of reviews (KQ3), the quality of systematic reviews will be evaluated using AMSTAR 2 [117]. We will rate the overall quality of a systematic review using the algorithm by Shea et al. [117]. If any of the seven critical AMSTAR 2 items are not met by a review, then we will judge the review to have a “critical flaw.” We will deem that the review has a “noncritical weakness” if any of the remaining noncritical items are not met. Any reviews with one or more critical flaws will receive a low or critically low rating, respectively. Reviews with a maximum of one noncritical weaknesses will be judged to be of high quality, and reviews with multiple noncritical weaknesses will be judged to be of moderate quality.

#### KQ1

For KQ1, the working group will rely on the study design-specific criteria used by the USPSTF which assigned a quality rating of “good,” “fair,” or “poor” [118]. Risk-of-bias assessments will only be conducted if studies excluded by the 2021 USPSTF systematic review are deemed to meet working group criteria and are included (e.g., French language publications).

### Synthesis of included studies

#### KQ1, KQ2, and KQ4

When synthesizing evidence included in our systematic reviews (KQ1, KQ2, KQ4), we will describe the study characteristics, participant characteristics, intervention and comparator details, outcome results, and risk-of-bias assessments for the included studies. Original study data may be converted to ensure consistent presentation and synthesis of the results across studies. We will present the relative risk or odds ratio with corresponding 95% confidence intervals. For calculating relative and absolute effects with 95% confidence intervals and absolute risk reduction for the summary of findings tables, we will follow GRADE guidance [119, 120]. If various measurement tools were used across studies, we will report the standardized mean difference with 95% confidence intervals. We will present the range of effects and follow guidance on narrative synthesis when describing the results narratively [121, 122]. Overdiagnosis rates will be extracted as defined and reported by study authors and descriptively analyzed or meta-analyzed if appropriate. In the absence of reported data, we will undertake our own calculations for overdiagnosis at the analysis stage. We may dichotomize individuals as being hypertensive or not or assign them a risk of future event. If hypertension is analyzed as a dichotomous outcome (i.e., present or absent), overdiagnosis will be calculated as the excess number of cases in the screening group over the total number of individuals screened, the number of individuals diagnosed with hypertension in the screening group, and per 1000 individuals screened, respectively. We will assess clinical (e.g., patient characteristics) and methodological (e.g., study design) heterogeneity of the included studies. Statistical heterogeneity will be assessed using the *I*^2^ statistic and Cochran’s Q test (threshold *p*-value < 0.10). We will consider the following levels of heterogeneity: low (0–25%), moderate (25–50%), substantial (50–75%), and considerable (> 75%) [123–127].

If pooling of the studies is appropriate following the heterogeneity assessments, we will pool the included studies using the DerSimonian and Laird random-effects method. We will pool data from randomized controlled trials and controlled clinical trials separately from observational studies. If considerable heterogeneity (> 75%) is detected [127], we may not pool the studies and will attempt to explain possible reasons for clinical heterogeneity through subgroup analyses and meta-regression.

Where possible, we will perform separate subgroup analyses according to the following:

- Gender/sex
- Type of intervention/screening method
- Setting
- Age
- Socioeconomic status
- Country/area of residence
- Race/ethnicity

To assess the robustness of our results, we may perform sensitivity analyses. This may include restricting analyses to studies only at low risk of bias, restricting by different types of publications (e.g., removing preprints), or restricting by issues considered in the risk-of-bias assessments (e.g., only including outcomes measured with validated measurement tools). Other considerations may become apparent during the conduct of the reviews that may require examination through sensitivity analyses. These additional considerations are deemed exploratory and should not be construed as a priori with a definitive hypothesis.

We will follow guidance based on random-effects models for meta-regression analyses and when we have at least 10 studies for outcome/intervention comparisons [91]. For assessing small-study effects (e.g., publication bias), we will use funnel plots and statistical tests (e.g., Egger regression test, Hedges-Olkin method, trim-and-fill method) [125, 128, 129].

For low event rates (less than 1%), we will use the Peto one-step odds ratio fixed-effect method [127]. The Mantel–Haenszel fixed-effect method will be used when group imbalances exist (e.g., control groups of unequal sizes), a large magnitude of the effect is observed, or when events are more frequent (5 to 10%) [130].

If any data or additional information is missing for our analyses, we will contact the corresponding authors of the study thrice by email over 1 month.

#### KQ3

For the overview of reviews (KQ3), we will present the characteristics and statistical outcomes reported in original reviews in tables, as well as a narrative summary of results. Review data may be converted to ensure consistent presentation and synthesis of the results, and, as needed, we will follow GRADE guidance to calculate relative and absolute risk differences from data reported in the reviews [119, 120]. We will present information from reviews that have undertaken subgroup/meta-regression analyses for the subgroup analyses factors described above. We will also note reviews with a focus on one of these factors in their scope (e.g., reviews blood pressure treatment initiation in adults over 50 years of age).

As an exploration of heterogeneity between overlapping systematic reviews, we will examine reasons for potential discordance using the algorithm Jadad et al. [131]. When the same primary studies are included in overlapping discordant reviews, we will examine the methodologic quality of the reviews, followed by issues in data extraction, heterogeneity testing, and methods of data synthesis in the reviews. When included primary studies differ among reviews that overlap in scope, we will investigate differences in eligibility criteria. Among reviews with the same selection criteria, this includes discordance that may be attributable in search strategies or application of selection criteria. When reviews differ in their eligibility criteria, we will explore differences in review publication status, methodologic quality of primary studies, language of review publication, and availability of patient-level data.

### Grading the certainty of evidence and interpretation

For the outcomes of interest, we will grade the certainty of evidence using the Grading of Recommendations, Assessment, Development and Evaluation (GRADE) approach [132, 133]. The GRADE framework involves rating (or grading) each of the following five domains for each outcome: study limitations (risk of bias), inconsistency or data heterogeneity, indirectness of evidence, imprecision of effect size estimates, and risk of publication (small study) bias. We will grade the five domains and then determine the overall certainty of the evidence for each outcome as either “very low,” “low,” “moderate,” or “high.” Trials (beginning at “high” certainty) and observational studies (beginning at “low” certainty) will be assessed separately.

#### KQ1

For KQ1, the working group will rely on the adapted approach by the USPSTF’s Evidence-Based Practice Center, which was based on the GRADE working group’s approach [89, 134]. This approach addresses four of the five GRADE framework domains: study limitations (risk of bias), inconsistency or data heterogeneity, imprecision of effect size estimates, and risk of publication (small study) bias. The USPSTF graded the overall strength of evidence as “high,” “moderate,” “low,” or “insufficient,” and their approach is further detailed in Additional file 9. For the working group to complete their evidence-to-decision (EtD) tables, we will address the omitted domain of indirectness of the evidence using our approach described above and revise the USPSTF overall GRADE ratings if necessary. Any modifications to the USPSTF grading will be reported in the final manuscript.

#### KQ2

For KQ2 (different BP measurement methods for prognosis), we will follow GRADE guidance on the assessment of evidence about prognostic factors [135]. As the best evidence for these this type of question is usually observational, these will begin at “high” certainty of evidence [135].

#### KQ4

For KQ4 (patient acceptability of screening), we will follow the GRADE guidance on grading the certainty of evidence on patient values and preferences [136, 137].

#### KQ3

For KQ3 (overview of reviews), we will provide GRADE assessments for the overall certainty of evidence for each outcome. For any systematic reviews included from the 2019 NICE review, we will rely on their GRADE assessments. Their modified approach is detailed in Additional file 8. For newly included systematic reviews, if the review authors have used GRADE methods, we will rely on their assessments for the overall quality of evidence, as well as ratings for each of the GRADE domains (i.e., risk of bias, imprecision, indirectness, inconsistency, publication bias). Primary studies will not be consulted to verify the GRADE ratings conducted in systematic reviews. If newly included reviews did not use GRADE methodology, GRADE assessments will be completed using information available from the review (e.g., risk-of-bias assessments). We may be limited by reporting issues in the systematic reviews, but we will provide our best interpretation and note any limitations we encounter in conducting the assessments using review data.

Before conducting the grading, reviewers will pilot GRADE assessments on a sample of five outcomes using GRADEpro GDT online software until reviewer agreement is high (i.e., at least four out of five domain ratings match). A senior team member will be consulted for any conflicts. The GRADE ratings will be performed independently and in duplicate by reviewers. A senior team member will be consulted for any disagreements.

For each critical and important outcome, we will create separate GRADE summary of findings tables with explanations for rating up or down for each domain [119, 120]. GRADE narrative statements will be used to communicate the findings and certainty of the evidence [120, 138, 139]. If a meta-analysis is not appropriate due to considerable heterogeneity, we will follow GRADE guidance on rating the certainty of evidence when there is no single estimate of effect [140]. Unless the outcome has a known minimally important difference around which to base our conclusions and certainty, we will initially apply a minimally contextualized approach, whereby we will rate certainty in the direction of effect (i.e., relative to the null effect) rather than a particular magnitude of effect. The minimally important difference will be discussed throughout the systematic review process and decided upon prior to the synthesis stage based on input from the working group, as informed by various potential sources (e.g., information from values/preferences studies). Upon examining the findings, the task force may decide to adopt a minimally contextualized approach using a threshold for small but important effect OR a partially contextualized approach using a range of magnitudes. In such case, we will revise ratings accordingly [141, 142]. Depending on the approach, we will rate our certainty on whether the true effect either lies on one side of the null threshold (i.e., that a non-null effect is present), on one side of a minimally important threshold (i.e., that there is an important versus trivial effect), or within ranges of specific magnitudes (i.e., no, or trivial, small, moderate, or large effect [141].

Grading of the certainty of evidence will be used in the subsequent GRADE EtD tables prepared by the working group and Science Team [143, 144]. In addition, EtD development will consider additional information beyond these planned systematic reviews (e.g., cost, feasibility) to assist the working group in developing updated clinical practice recommendations. Details on the Task Force guideline development process is available in their Methods Manual (note: currently under revision) [91].

### Reporting

The de novo systematic reviews will be reported using PRISMA (KQ2 and KQ4) [108], and overview of reviews (KQ3) will be reported using the Preferred Reporting Items for Overviews of systematic reviews including harms pilot checklist (PRIO-harms) [145].

## Discussion

Hypertension is a leading risk factor for cardiovascular morbidity and death in Canada and worldwide, affecting over 20% of Canadian adults. Hypertension screening can provide a benefit when previously untreated hypertension is diagnosed and brought under control, but the potential for harm must be considered. There is a need for updated recommendations on optimal screening methods, screening frequency, target population, and patient values and preferences. Since the release of the 2012 Task Force guideline on screening for hypertension in adults [86], the previous key questions require updating, and additional key questions have been developed. Findings from the planned systematic reviews will inform the Task Force on the update of their recommendations for hypertension screening in adults.

### Supplementary Information

**Additional file 1.** 2012 CHEP Recommendations for Accurate Measurement of BP [85].

**Additional file 2.** PRISMA-P 2015 checklist.

**Additional file 3.** AMSTAR 2 ratings for USPSTF 2021 systematic review.

**Additional file 4.** Draft search strategies.

**Additional file 5.** PRESS checklist.

**Additional file 6.** List of grey literature relevant websites.

**Additional file 7.** Draft data extraction items.

**Additional file 8.** UK NICE grading the strength of the body of evidence.

**Additional file 9.** USPSTF grading the strength of the body of evidence.

**Additional file 10.** Stakeholder review and feedback.

## Data Availability

Not applicable.

## Abbreviations

ABPM: Ambulatory blood pressure measurement
BP: Blood pressure
CCT: Controlled clinical trial
CVD: Cardiovascular disease
DBP: Diastolic blood pressure
ERSC: Evidence Review and Synthesis Centre
GRADE: Grading of Recommendation Assessment, Development and Evaluation
HBPM: Home blood pressure measurement
KQ: Key question
NICE: National Institute for Health and Care Excellence
OBPM: Office blood pressure measurement
RCT: Randomized controlled trial
SBP: Systolic blood pressure
USPSTF: United States Preventive Services Task Force
