# Supplementary material for: Screening for hypertension in adults: protocol for evidence reviews to inform a Canadian Task Force on Preventive Health Care guideline update
Source: Syst Rev. 2024 Jan 5;13:17. doi: 10.1186/s13643-023-02392-1 (PMC10768239; doi:10.1186/s13643-023-02392-1)
Supplement: Supplementary file 1 — Additional file 1. 2012 CHEP Recommendations for Accurate Measurement of BP [85]. [file 13643_2023_2392_MOESM1_ESM.docx]

## **Additional file 1: 2012 CHEP Recommendations for Accurate Measurement of BP** [85]

Recommendations

1. Health care professionals who have been specifically trained to measure BP accurately should assess BP in all adult patients at all appropriate visits to determine cardiovascular risk and monitor antihypertensive treatment (Grade D).

2. Use of standardized measurement techniques is recommended when assessing BP (Grade D):


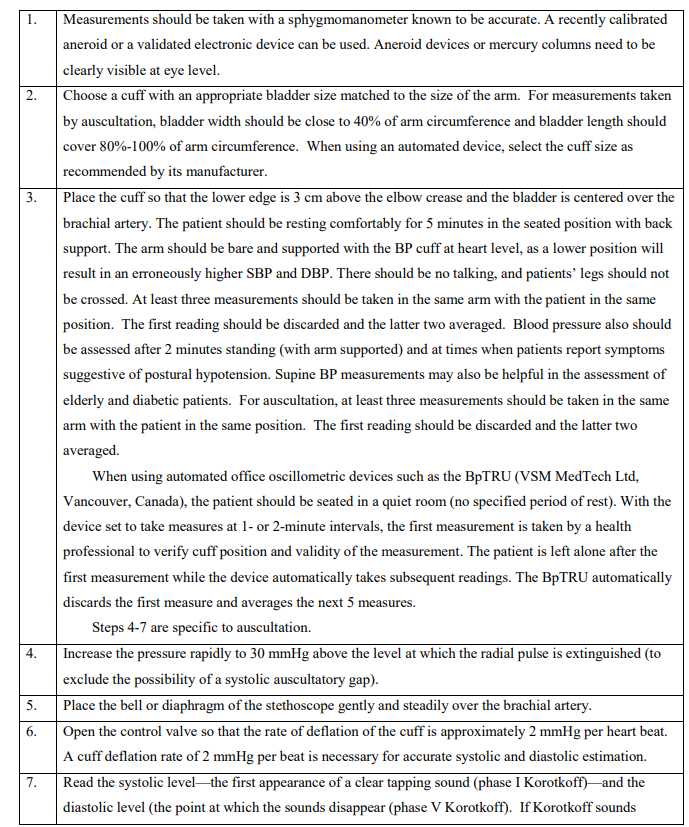


3. Automated office BP measurements (OBPM) can be used in the assessment of office BP (Grade D).

4. When used in proper conditions, automated office systolic BP (SBP) of ≥135 mm Hg or diastolic BP (DBP) of ≥85mm Hg should be considered analogous to mean awake ambulatory SBP of ≥135 mm Hg and DBP of ≥85 mmHg, respectively (Grade D).
