# Supplementary material for: Screening for hypertension in adults: protocol for evidence reviews to inform a Canadian Task Force on Preventive Health Care guideline update
Source: Syst Rev. 2024 Jan 5;13:17. doi: 10.1186/s13643-023-02392-1 (PMC10768239; doi:10.1186/s13643-023-02392-1)
Supplement: Supplementary file 3 — Additional file 3. AMSTAR 2 ratings for USPSTF 2021 systematic review. [file 13643_2023_2392_MOESM3_ESM.docx]

## **Additional file 3: AMSTAR 2 ratings for USPSTF 2021 systematic review**

| **Author; Year** | **AMSTAR 2 Items** | | | | | | | | | | | | | | | | | | **Overall AMSTAR Rating** |
| --- | --- | --- | --- | --- | --- | --- | --- | --- | --- | --- | --- | --- | --- | --- | --- | --- | --- | --- | --- |
|  | **1** | **2** | **3** | **4** | **5** | **6** | **7** | **8** | **9** | | **10** | **11** | | **12** | **13** | **14** | **15** | **16** |  |
|  |  |  |  |  |  |  |  |  | **a** | **b** |  | **a** | **b** |  |  |  |  |  |  |
| USPSTF; 2021 [89] | Yes | Yes | Yes | Yes | No | NR | Partial yes^1^ | Yes | Yes | Yes | No^2^ | Yes | N/A | No^3^ | Yes | Yes | Yes | Yes | High |

^1. The excluded studies list was reported, along with reasons for exclusion. However, the review excluded low quality studies, rather than assessing the effect of low-quality studies on the certainty of evidence.^

^2. Review authors did not report on the sources of funding for the studies included in the review.^

^3. Review authors did not assess the potential impact of RoB in individual studies on the results of the meta-analysis, as low RoB studies were excluded from the review.^

**Citation**: Guirguis-Blake JM, Evans CV, Webber EM, Coppola EL, Perdue LA, Weyrich MS (2020) Screening for Hypertension in Adults: A Systematic Evidence Review for the U.S. Preventive Services Task Force. <https://www.uspreventiveservicestaskforce.org/uspstf/document/draft-evidence-review/hypertension-in-adults-screening>.
