# Supplementary material for: Screening for hypertension in adults: protocol for evidence reviews to inform a Canadian Task Force on Preventive Health Care guideline update
Source: Syst Rev. 2024 Jan 5;13:17. doi: 10.1186/s13643-023-02392-1 (PMC10768239; doi:10.1186/s13643-023-02392-1)
Supplement: Supplementary file 4 — Additional file 4. Draft search strategies. [file 13643_2023_2392_MOESM4_ESM.docx]

## **Additional file 4: Draft search strategies**

### **Key question 2**

Database: Ovid MEDLINE(R) ALL <1946 to July 01, 2022>

Search Strategy:

--------------------------------------------------------------------------------

1 Blood Pressure/ (287662)

2 (blood pressure? or (arter* adj2 pressure?) or (aortic* adj2 pressure?) or (systolic* adj2 pressure?) or (diastolic* adj2 pressure?)).ti,kw,kf. (96358)

3 ((arter* or aortic* or systolic* or diastolic*) adj BP).ti,kw,kf. (80)

4 or/1-3 [BLOOD PRESSURE] (316041)

5 Reference Standards/ (45316)

6 reference standard*.ti,kw,kf. (1343)

7 Reference Values/ (163403)

8 (reference adj (limit? or value? or range or ranges)).ti,kw,kf. (6800)

9 (normal level? or normal limit* or normal range? or normal value?).ti,kw,kf. (4213)

10 or/5-9 [REFERENCE STANDARDS/VALUES] (211308)

11 4 and 10 [BLOOD PRESSURE - REFERENCE STANDARDS/VALUES] (6802)

12 Blood Pressure Determination/ (29439)

13 Blood Pressure Monitoring, Ambulatory/ (11224)

14 exp Sphygmomanometers/ (3298)

15 (blood pressure? adj3 (assess* or determin* or level? or measur* or monitor* or rang* or status* or target* or telemonitor* or tele-monitor* or threshold? or value?)).ti,kw,kf. (14146)

16 (arter* adj2 pressure? adj3 (assess* or determin* or level? or measur* or monitor* or rang* or status* or target* or telemonitor* or tele-monitor* or threshold? or value?)).ti,kw,kf. (1637)

17 (aortic* adj2 pressure? adj3 (assess* or determin* or level? or measur* or monitor* or rang* or status* or target* or telemonitor* or tele-monitor* or threshold? or value?)).ti,kw,kf. (117)

18 (systolic* adj2 pressure? adj3 (assess* or determin* or level? or measur* or monitor* or rang* or status* or target* or telemonitor* or tele-monitor* or threshold? or value?)).ti,kw,kf. (414)

19 (diastolic* adj2 pressure? adj3 (assess* or determin* or level? or measur* or monitor* or rang* or status* or target* or telemonitor* or tele-monitor* or threshold? or value?)).ti,kw,kf. (103)

20 (BP adj3 (assess* or determin* or level? or measur* or monitor* or rang* or status* or target* or telemonitor* or tele-monitor* or threshold? or value?)).ti,kw,kf. (241)

21 (AMBP adj3 (assess* or determin* or level? or measur* or monitor* or rang* or status* or target* or telemonitor* or tele-monitor* or threshold? or value?)).ti,kw,kf. (2)

22 ((OBPM or AOBP) adj10 (pressure? or BP)).ti,kw,kf. (7)

23 sphygmomanometer*.ti,kw,kf. (512)

24 or/12-23 [BLOOD PRESSURE MEASUREMENT] (46195)

25 11 or 24 [BLOOD PRESSURE MEASUREMENT/REFERENCE VALUES/STANDARDS] (51672)

26 Cardiovascular Diseases/ (168348)

27 exp *Cardiovascular Diseases/ (2262037)

28 ((artery or arteries or arterial or atrial* or atrium* or cardiac* or cardio* or coronary or heart? or myocard* or ventricle*) adj3 (arrest* or aneurysm* or arrhythmia* or arrythmia* or disease* or disorder* or dysrhythmia* or dysrythmia* or dysfunction* or failure* or fibrillation* or ischemi* or infarction* or obstruct* or rupture* or syndrome*)).ti,kw,kf. (588813)

29 (arterioscleros* or arterio-scleros* or atheroscleros* or athero-scleros*).ti,kw,kf. (64765)

30 (isch?emi* adj2 stroke?).ti,kw,kf. (35330)

31 (CVD and (artery or arteries or arterial or atrial* or atrium* or cardiac* or cardio* or coronary or heart? or myocard* or ventricle*)).ti,kw,kf. (1734)

32 (CHD and (artery or arteries or arterial or atrial* or atrium* or cardiac* or cardio* or coronary or heart* or myocard* or ventricle*)).ti,kw,kf. (1546)

33 or/26-32 [CVD] (2406535)

34 Hypertension/ (250551)

35 Essential Hypertension/ (2598)

36 Masked Hypertension/ (441)

37 White Coat Hypertension/ (531)

38 Prehypertension/ (1105)

39 hypertensi*.ti,kw,kf. (236763)

40 (prehypertensi* or pre-hypertensi*).ti,kw,kf. (1452)

41 (blood pressure? adj3 (elevat* or high* or increas* or rais?)).ti,kw,kf. (9198)

42 (arter* adj2 pressure? adj3 (elevat* or high* or increas* or rais?)).ti,kw,kf. (744)

43 (aortic* adj2 pressure? adj3 (elevat* or high* or increas* or rais?)).ti,kw,kf. (47)

44 (systolic* adj2 pressure? adj3 (elevat* or high* or increas* or rais?)).ti,kw,kf. (292)

45 (diastolic* adj2 pressure? adj3 (elevat* or high* or increas* or rais?)).ti,kw,kf. (107)

46 or/34-45 [HYPERTENSION] (343636)

47 33 or 46 [CVD, INCL. HYPERTENSION] (2491861)

48 25 and 47 [BLOOD PRESSURE MEASUREMENT/REFERENCE VALUES/STANDARDS - CVDs, INCL. HYPERTENSION] (30261)

49 exp Risk Assessment/ (304467)

50 Risk Factors/ (926997)

51 Heart Disease Risk Factors/ (3780)

52 Forecasting/ (90407)

53 Morbidity/td [Trends] (5902)

54 Mortality/td [Trends] (11206)

55 Survival Rate/td [Trends] (16036)

56 Probability/ (59355)

57 Prognosis/ (576275)

58 Bayes Theorem/ (43746)

59 (bayes theor* or bayesian).ti,kw,kf. (23243)

60 exp Regression Analysis/ (456002)

61 regression*.ti,kw,kf. (45149)

62 (risk? adj3 (assess* or calculat* or deriv* or determin* or factor* or measur* or monitor*)).ti,kw,kf. (220089)

63 ((forecast* or future or predict* or probabilit* or prognos* or trend*) adj5 (death* or disease* or event* or morbidit* or mortalit*)).ti,kw,kf. (54797)

64 or/49-63 [RISK] (2215827)

65 48 and 64 (7061) [RISK - BLOOD PRESSURE MEASUREMENT/REFERENCE VALUES/STANDARDS - CVDs, INCL. HYPERTENSION]

66 exp Child/ not (exp Adult/ or Adolescent/) (908793)

67 exp Infant/ not (exp Adult/ or Adolescent/) (758090)

68 65 not (66 or 67) [CHILD-, INFANT-ONLY REMOVED] (6915)

69 (controlled clinical trial or randomized controlled trial or pragmatic clinical trial or equivalence trial).pt. (663555)

70 "Clinical Trials as Topic"/ (200137)

71 exp "Controlled Clinical Trials as Topic"/ (165833)

72 (randomi#ed or randomi#ation? or randomly or RCT or placebo*).tw,kw,kf. (1140356)

73 ((singl* or doubl* or trebl* or tripl*) adj (mask* or blind* or dumm*)).tw,kw,kf. (189909)

74 trial.ti. (265580)

75 or/69-74 [RCT FILTER] (1593116)

76 68 and 75 [RCTs] (977)

77 exp Cohort Studies/ (2367121)

78 cohort$1.tw,kw,kf. (766659)

79 Retrospective Studies/ (1040292)

80 (longitudinal* or prospective* or retrospective*).tw,kw,kf. (1925273)

81 ((followup or follow-up) adj (study or studies)).tw,kw,kf. (56490)

82 Observational study.pt. (129389)

83 (observation$2 adj (study or studies)).tw,kw,kf. (145169)

84 ((population or population-based) adj (study or studies or analys#s)).tw,kw,kf. (26681)

85 ((multidimensional or multi-dimensional) adj (study or studies)).tw,kw,kf. (141)

86 Comparative Study.pt. (1911253)

87 ((comparative or comparison) adj (study or studies)).tw,kw,kf. (127647)

88 Cross-Sectional Studies/ (431939)

89 (crosssection* or cross section*).tw,kw,kf. (501162)

90 Case-Control Studies/ (322097)

91 ((case-control* or case-comparison or case-compeer or case-refer?ent or case-base) adj3 (stud* or design?)).tw,kw,kf. (136473)

92 or/77-91 [OBSERVATIONAL/COHORT FILTER] (5586656)

93 68 and 92 [OBSERVATIONAL/COHORT STUDIES] (4162)

94 76 or 93 [RCTs, OBSERVATIONAL/COHORT STUDIES] (4556)

95 exp Animals/ not Humans/ (5023830)

96 94 not 95 [ANIMAL-ONLY REMOVED] (4515)

97 (comment or editorial or news or newspaper article).pt. (1599768)

98 (letter not (letter and randomized controlled trial)).pt. (1179752)

99 96 not (97 or 98) [OPINION PIECES REMOVED] (4415)

100 limit 99 to english (4137)

101 limit 99 to french (52)

102 100 or 101 [LANGUAGE LIMITS] (4188)

***************************

### **Key question 3**

Database: Ovid MEDLINE(R) ALL <1946 to July 12, 2022>

Search Strategy:

--------------------------------------------------------------------------------

1 Hypertension/ (250808)

2 Essential Hypertension/ (2606)

3 Masked Hypertension/ (442)

4 White Coat Hypertension/ (533)

5 Prehypertension/ (1108)

6 hypertens*.tw,kw,kf. (488586)

7 (prehypertensi* or pre-hypertensi*).tw,kw,kf. (3633)

8 (blood pressure? adj3 (elevat* or high* or increas* or rais?)).tw,kw,kf. (68869)

9 (arter* adj2 pressure? adj3 (elevat* or high* or increas* or rais?)).tw,kw,kf. (18911)

10 (aortic* adj2 pressure? adj3 (elevat* or high* or increas* or rais?)).tw,kw,kf. (994)

11 (systolic* adj2 pressure? adj3 (elevat* or high* or increas* or rais?)).tw,kw,kf. (12726)

12 (diastolic* adj2 pressure? adj3 (elevat* or high* or increas* or rais?)).tw,kw,kf. (7456)

13 (BP adj3 (elevat* or high* or increas* or rais?)).tw,kw,kf. (16647)

14 (AMBP adj3 (elevat* or high* or increas* or rais?)).tw,kw,kf. (31)

15 or/1-14 [HYPERTENSION] (591247)

16 (exp Child/ or exp Infant/) not (Adolescent/ or exp Adult/) (1398583)

17 15 not 16 [CHILD-, INFANT-ONLY REMOVED] (572611)

18 Hypertension/dt [drug therapy] (66776)

19 Essential Hypertension/dt [drug therapy] (154)

20 Masked Hypertension/dt [drug therapy] (58)

21 White Coat Hypertension/dt [drug therapy] (64)

22 Prehypertension/dt [drug therapy] (102)

23 exp Antihypertensive Agents/ (267062)

24 (anti-hypertens* or antihypertens* or anti hypertens*).tw,kw,kf. (59124)

25 exp Angiotensin-Converting Enzyme Inhibitors/ (46460)

26 ACE inhibitor?.tw,kw,kf. (18687)

27 (angiotensin adj2 converting enzyme adj3 (inhibitor? or antagonist?)).tw,kw,kf. (28645)

28 (ACEI or ACEIs).tw,kw,kf. (5519)

29 (Kininase II adj1 (inhibitor? or antagonist?)).tw,kw,kf. (74)

30 exp Angiotensin Receptor Antagonists/ (26842)

31 (angiotensin adj4 receptor adj (block? or antagonist?)).tw,kw,kf. (4432)

32 (sartan$2 or sartans$2).tw,kw,kf. (402)

33 ((ARB or ARBs) and angiotensin*).tw,kw,kf. (6449)

34 Calcium Channel Blockers/ (37457)

35 (calcium adj2 (antagonist* or blocker* or blocking or blockade* or inhibitor*)).tw,kw,kf. (36249)

36 Amlodipine/ (4064)

37 (amlodipine$2 or amlodis$2 or amlor$2 or astudal$2 or istin$2 or norvasc$2).tw,kw,kf. (5530)

38 Felodipine/ (1256)

39 (felodipine$2 or felodipin$2 or agon$2 or felo biochemie$2 or felo-puren$2 or felobeta$2 or felocor$2 or felodur$2 or felogamma$2 or fensel$2 or flodil$2 or modip$2 or munobal$2 or perfudal$2 or plendil$2 or renedil$2).tw,kw,kf. (3542)

40 Nifedipine/ (15770)

41 (nifedipine$2 or adalat$2 or "BAY-a-1040" or "Bay-1040" or cordipin$2 or cordipine$2 or corinfar$2 or fenigidin$2 or korinfar$2 or nifangin$2 or procardia$2 or Vascard$2).tw,kw,kf. (20119)

42 Diltiazem/ (6260)

43 (diltiazem$2 or aldizem$2 or "CRD-401" or cardil$2 or cardizem$2 or dilacor$2 or dilren$2 or dilzem$2 or tiazac$2).tw,kw,kf. (8859)

44 exp Verapamil/ (17699)

45 (verapamil$2 or calan$2 or cordilox$2 or dexverapamil$2 or falicard$2 or finoptin$2 or iproveratril$2 or isoptin$2 or isoptine$2 or izoptin$2 or lekoptin$2).tw,kw,kf. (23701)

46 exp Diuretics/ (82409)

47 diuretic*.tw,kw,kf. (42393)

48 (sodium chloride adj2 inhibitor?).tw,kw,kf. (28)

49 (NaCl adj2 inhibitor?).tw,kw,kf. (84)

50 Indapamide/ (1042)

51 (indapamide$2 or metindamide$2 or "S-1520" or "SE-1520").tw,kw,kf. (1269)

52 Bendroflumethiazide/ (641)

53 (bendroflumethiazide$2 or aprinox$2 or bendrofluazide$2 or benzide$2 or berkozide$2 or centyl$2 or esberizid$2 or naturetin$2 or naturine$2 or neo-naclex$2 or pluryl$2 or urizid$2).tw,kw,kf. (612)

54 exp Adrenergic beta-Antagonists/ (86392)

55 (betablock? or beta-block*).tw,kw,kf. (39334)

56 (beta adj1 (adrenergic or adrenoceptor?) adj3 (antagonist* or block*)).tw,kw,kf. (16978)

57 or/18-56 [ANTI-HYPERTENSIVES] (497586)

58 17 and 57 [HYPERTENSION - DRUG THERAPY] (134168)

59 exp Risk Assessment/ (304602)

60 Risk Factors/ (927910)

61 Heart Disease Risk Factors/ (3816)

62 Forecasting/ (90446)

63 Morbidity/td [Trends] (5902)

64 Mortality/td [Trends] (11206)

65 Survival Rate/td [Trends] (16036)

66 Probability/ (59390)

67 Prognosis/ (576816)

68 Bayes Theorem/ (43909)

69 (bayes theor* or bayesian).ti,kf. (23344)

70 exp Regression Analysis/ (456126)

71 regression*.ti,kf. (45320)

72 (risk? adj3 (assess* or calculat* or deriv* or determin* or factor* or measur* or monitor*)).tw,kw,kf. (898437)

73 ((forecast* or future or predict* or probabilit* or prognos* or trend*) adj5 (death* or disease* or event* or morbidit* or mortalit*)).tw,kw,kf. (272804)

74 (risk* adj2 (factor* or assess*)).tw,kw,kf. (826055)

75 ((cardiovascular or cardio-vascular or CVD) adj3 risk?).tw,kw,kf. (142509)

76 ((initiat* or start* or commenc* or begin*) adj4 (medicat* or therap* or treatment*)).tw,kw,kf. (168259)

77 (threshold* or level*).tw,kw,kf. (4775698)

78 or/59-77 [RISK] (7042030)

79 58 and 78 [HYPERTENSION - DRUG THERAPY - RISK] (50346)

80 exp Animals/ not Humans/ (5027170)

81 79 not 80 [ANIMAL-ONLY REMOVED] (43618)

82 (editorial or news or newspaper article).pt. (841280)

83 ((case report or comment or letter) not randomized controlled trial).pt. (1652916)

84 (case report or comment or letter).pt. not random*.ti. (1648202)

85 or/82-84 (2288784)

86 81 not 85 [OPINION PIECES, CASE REPORTS REMOVED] (42479)

87 Systematic Review.pt. (201473)

88 exp Systematic Reviews as Topic/ (8695)

89 Meta Analysis.pt. (163898)

90 exp Meta-Analysis as Topic/ (25262)

91 (meta-analy* or metanaly* or metaanaly* or met analy* or integrative research or integrative review* or integrative overview* or research integration or research overview* or collaborative review*).tw,kw,kf. (246957)

92 (systematic review* or systematic overview* or evidence-based review* or evidence-based overview* or (evidence adj3 (review* or overview*)) or meta-review* or meta-overview* or meta-synthes* or mapping review? or rapid review* or "review of reviews" or scoping review? or umbrella review? or technology assessment* or HTA or HTAs).tw,kw,kf. (323211)

93 exp Technology Assessment, Biomedical/ (11926)

94 (cochrane or health technology assessment or evidence report or systematic reviews).jw. (21491)

95 Network Meta-Analysis/ (3917)

96 (network adj (MA or MAs)).tw,kw,kf. (17)

97 (NMA or NMAs or MTC or MTCs or MAIC or MAICs).tw,kw,kf. (8698)

98 indirect* compar*.tw,kw,kf. (2537)

99 (indirect treatment* adj1 compar*).tw,kw,kf. (411)

100 (mixed treatment* adj1 compar*).tw,kw,kf. (513)

101 (multiple treatment* adj1 compar*).tw,kw,kf. (218)

102 (multi-treatment* adj1 compar*).tw,kw,kf. (2)

103 simultaneous* compar*.tw,kw,kf. (1242)

104 mixed comparison?.tw,kw,kf. (41)

105 or/87-104 [REVIEW FILTER] (501670)

106 86 and 105 [REVIEWS] (1982)

107 (2018* or 2019* or 202*).dt. (6431477)

108 106 and 107 [SRs - UPDATE PERIOD] (572)

***************************

### **Key question 4**

Database: Ovid MEDLINE(R) ALL <1946 to July 01, 2022>

Search Strategy:

--------------------------------------------------------------------------------

1 Hypertension/ (250551)

2 Essential Hypertension/ (2598)

3 Masked Hypertension/ (441)

4 White Coat Hypertension/ (531)

5 Prehypertension/ (1105)

6 hypertensi*.tw,kw,kf. (487635)

7 (prehypertensi* or pre-hypertensi*).tw,kw,kf. (3625)

8 (blood pressure? adj3 (elevat* or high* or increas* or rais?)).tw,kw,kf. (68783)

9 (aortic* adj2 pressure? adj3 (elevat* or high* or increas* or rais?)).tw,kw,kf. (995)

10 (arter* adj2 pressure? adj3 (elevat* or high* or increas* or rais?)).tw,kw,kf. (18903)

11 (systolic* adj2 pressure? adj3 (elevat* or high* or increas* or rais?)).tw,kw,kf. (12712)

12 (diastolic* adj2 pressure? adj3 (elevat* or high* or increas* or rais?)).tw,kw,kf. (7447)

13 (BP adj3 (elevat* or high* or increas* or rais?)).tw,kw,kf. (16614)

14 ((AMBP or OBPM or AOBP) adj3 (elevat* or high* or increas* or rais?)).tw,kw,kf. (64)

15 or/1-14 [HYPERTENSION] (590258)

16 Mass Screening/ (113889)

17 (screen* or detect*).tw,kw,kf. (3327971)

18 (identif* or recogni*).ti. (435645)

19 ((early or earlier or earliest) adj5 (identif* or recogni*)).tw,kw,kf. (83503)

20 (case finding? or casefinding?).tw,kw,kf. (6080)

21 or/16-20 [SCREENING] (3739370)

22 15 and 21 [HYPERTENSION SCREENING] (61161)

23 exp Child/ not (exp Adult/ or Adolescent/) (908793)

24 exp Infant/ not (exp Adult/ or Adolescent/) (758090)

25 22 not (23 or 24) [CHILD-, INFANT-ONLY REMOVED] (59224)

26 exp Animals/ not Humans/ (5023830)

27 25 not 26 [ANIMAL-ONLY REMOVED] (54188)

28 (comment or editorial or news or newspaper article).pt. (1599768)

29 (letter not (letter and randomized controlled trial)).pt. (1179752)

30 27 not (28 or 29) [OPINION PIECES REMOVED] (53547)

31 Choice Behavior/ (34518)

32 (choice? adj2 behavio?r*).tw,kw,kf. (3506)

33 Cooperative Behavior/ (45659)

34 Decision Making/ (102783)

35 decision aid*.tw,kw,kf. (4108)

36 ((guide? or guiding or make or making or makes or made or shar* or support*) adj2 (choice? or choos* or consent* or decid* or decision*)).tw,kw,kf. (245716)

37 Patient Education as Topic/ (88082)

38 Patient Acceptance of Health Care/ (53489)

39 Patient Participation/ (28717)

40 Patient Preference/ (10333)

41 Patient Satisfaction/ (87982)

42 ((engag* or involvement* or participat*) adj3 (patient? or person$2 or personally or man or men or "man's" or "men's" or wom#n or "woman's" or "women's")).tw,kw,kf. (96976)

43 exp Patients/px [psychology] (18380)

44 Uncertainty/ (16193)

45 ((accept* or consider* or choice? or choos* or chose? or decid* or decis* or expect* or input* or knowledge* or opinion* or participat* or perspective? or prefer* or respons* or satisf* or uncertain* or understand* or willing*) adj2 (female? or male? or man or men or "man's" or "men's" or patient? or person$2 or personally or wom#n or "woman's" or "women's")).tw,kw,kf. (402368)

46 ((analys#s or valuation? or value? or valuing) adj2 (conjoint or contingent)).tw,kw,kf. (2020)

47 (choice? adj1 (discrete or experiment*)).tw,kw,kf. (4519)

48 ((patient? or person$2 or personally or man or men or "man's" or "men's" or wom#n or "woman's" or "women's") adj (centered or centred or focus*)).tw,kw,kf. (42574)

49 ((patient* or person#2) adj priorit*).tw,kw,kf. (1127)

50 Informed Consent/ (38248)

51 (informed adj (choice* or choos* or consent* or decid* or decision*)).tw,kw,kf. (54928)

52 ((patient? or person$2 or personally or man or men or "man's" or "men's" or wom#n or "woman's" or "women's") adj2 consent*).tw,kw,kf. (13084)

53 ((adher* or nonadher* or non-adher* or inten* or refus* or reject* or uptake or willing*) adj2 (initiat* or intervention? or therap* or treat*)).tw,kw,kf. (120439)

54 (preference? adj1 (elicit* or reveal* or scor* or stated)).tw,kw,kf. (2484)

55 (trade off? or tradeoff? or trade-off?).tw,kw,kf. (37075)

56 Information Seeking Behavior/ (3063)

57 (inform* adj1 seek*).tw,kw,kf. (5679)

58 or/31-57 [PATIENT ACCEPTABILITY/DECISION-MAKING/PARTICIPATION] (1217317)

59 30 and 58 [HYPERTENSION SCREENING - PATIENT ACCEPTABILITY/DECISION-MAKING/PARTICIPATION] (3430)

60 (controlled clinical trial or randomized controlled trial or pragmatic clinical trial or equivalence trial).pt. (663555)

61 "Clinical Trials as Topic"/ (200137)

62 exp "Controlled Clinical Trials as Topic"/ (165833)

63 (randomi#ed or randomi#ation? or randomly or RCT or placebo*).tw,kw,kf. (1140356)

64 ((singl* or doubl* or trebl* or tripl*) adj (mask* or blind* or dumm*)).tw,kw,kf. (189909)

65 trial.ti. (265580)

66 or/60-65 (1593116)

67 59 and 66 [RCTs] (653)

68 controlled clinical trial.pt. (94928)

69 Controlled Clinical Trial/ or Controlled Clinical Trials as Topic/ (100520)

70 (control* adj2 trial).tw,kw,kf. (194003)

71 Non-Randomized Controlled Trials as Topic/ (1047)

72 (nonrandom* or non-random* or quasi-random* or quasi-experiment*).tw,kw,kf. (67468)

73 (nRCT or non-RCT).tw,kw,kf. (437)

74 Controlled Before-After Studies/ (699)

75 (control* adj3 ("before and after" or "before after")).tw,kw,kf. (4960)

76 Interrupted Time Series Analysis/ (1656)

77 time series.tw,kw,kf. (40669)

78 (pre- adj3 post-).tw,kw,kf. (103392)

79 (pretest adj3 posttest).tw,kw,kf. (6755)

80 Historically Controlled Study/ (222)

81 (control* adj2 study).tw,kw,kf. (198284)

82 Control Groups/ (1835)

83 (control* adj2 group?).tw,kw,kf. (582267)

84 trial.ti. (265580)

85 or/68-84 (1280670)

86 59 and 85 [NON-RCTs] (492)

87 exp Cohort Studies/ (2367121)

88 cohort?.tw,kw,kf. (766659)

89 Retrospective Studies/ (1040292)

90 (longitudinal or prospective or retrospective).tw,kw,kf. (1544000)

91 ((followup or follow-up) adj (study or studies)).tw,kw,kf. (56490)

92 Observational study.pt. (129389)

93 (observation$2 adj (study or studies)).tw,kw,kf. (145169)

94 ((population or population-based) adj (study or studies or analys#s)).tw,kw,kf. (26681)

95 ((multidimensional or multi-dimensional) adj (study or studies)).tw,kw,kf. (141)

96 Comparative Study.pt. (1911253)

97 ((comparative or comparison) adj (study or studies)).tw,kw,kf. (127647)

98 exp Case-Control Studies/ (1334049)

99 ((case-control* or case-based or case-comparison) adj (study or studies)).tw,kw,kf. (124109)

100 or/87-99 (5056862)

101 59 and 100 [OBSERVATIONAL STUDIES] (1310)

102 67 or 86 or 101 [RCTs, nRCTs, OBSERVATIONAL STUDIES] (1739)

103 limit 102 to english (1641)

104 limit 102 to french (16)

105 103 or 104 [LANGUAGE LIMITS] (1656)

***************************
