# Supplementary material for: Screening for hypertension in adults: protocol for evidence reviews to inform a Canadian Task Force on Preventive Health Care guideline update
Source: Syst Rev. 2024 Jan 5;13:17. doi: 10.1186/s13643-023-02392-1 (PMC10768239; doi:10.1186/s13643-023-02392-1)
Supplement: Supplementary file 6 — Additional file 6. List of grey literature relevant websites. [file 13643_2023_2392_MOESM6_ESM.docx]

## **Additional file 6: List of grey literature relevant websites**

| **Organization** | **Website Link** |
| --- | --- |
| **Relevant websites suggested by WG and clinical experts** |  |
| American Academy of Family Physicians | <https://www.aafp.org/> |
| American College of Physicians | <https://www.acponline.org/> |
| American Nurses Association | <https://www.nursingworld.org/> |
| Canadian Nurses Association | <https://www.cna-aiic.ca/> |
| College of Family Physicians of Canada | <https://www.cfp.ca/> |
| Hypertension Canada | <https://hypertension.ca/> |
| **Selected relevant CADTH sources** |  |
| **Canadian health technology and assessment agencies** |  |
| Alberta College of Family Physicians (ACFP) | <https://acfp.ca/> |
| CADTH | <https://www.cadth.ca/search?keywords> |
| Manitoba Centre for Health Policy (MCHP) | <http://mchp-appserv.cpe.umanitoba.ca/deliverablesList.html> |
| Ottawa Hospital Research Institute (OHRI) | <http://www.ohri.ca/ksgroup/Publications.aspx> |
| UBC Centre for Health Services and Policy Research | <https://chspr.ubc.ca/publications/> |
| **International health technology and assessment agencies** |  |
| INAHTA | <https://www.inahta.org/publications/> |
| WHO Health Evidence Network (HEN) | <https://www.euro.who.int/en/data-and-evidence/evidence-informed-policy-making/publications/by-keyword> |
| COAG Health Council | <https://www.coaghealthcouncil.gov.au/AHMAC/Health-Technology-Reference-Group/Reports-and-Briefs> |
| Belgian Health Care Knowledge Centre (KCE) | <https://kce.fgov.be/en/all-reports> |
| CEDIT Recommendations and Reports | <http://cedit.aphp.fr/cedit-hta-agency/recommendations-reports/> |
| French National Authority for Health (HAS) | <http://www.has-sante.fr/jcms/c_946986/en/english-toutes-nos-publications-ligne-principale?portal=r_1457306> |
| German Institute of Medical Documentation and Information (DIMDI) | <https://www.dimdi.de/dynamic/en/homepage/> |
| Health Information and Quality Authority | <https://www.hiqa.ie/reports-and-publications/health-technology-assessments> |
| Health Service Executive (Irish Health Repository) | <https://www.lenus.ie/hse/> |
| Health Council of the Netherlands | <https://www.gezondheidsraad.nl/> |
| National Health Care Institute Netherlands | <https://english.zorginstituutnederland.nl/publications> |
| Folkehelseinstituttet. Norwegian Institute of Public Health | <https://www.fhi.no/en/publ/> |
| Institute of Health Carlos III | <https://publicaciones.isciii.es/> |
| Healthcare Improvement Scotland | <http://www.healthcareimprovementscotland.org/> |
| NICE (NHS National Institute for Health and Care Excellence) | <https://www.nice.org.uk/> |
| NICE (Advice List) | <https://www.nice.org.uk/guidance/published> |
| NIHR | <http://www.io.nihr.ac.uk/> |
| NETSCC | <https://www.journalslibrary.nihr.ac.uk/programmes/> |
| National Health Service UK (NHS) England | <https://www.england.nhs.uk/> |
| AHRQ | <https://www.ahrq.gov/cpi/about/index.html> |
| Centers for Medicare and Medicaid Services (CMS) | <https://www.cms.gov/About-CMS/About-CMS> |
| Washington State Health Care Authority (HCA) | <https://www.hca.wa.gov/about-hca> |
| **Clinical Practice Guidelines** |  |
| Alberta Medical Association | <https://actt.albertadoctors.org/Pages/default.aspx> |
| British Columbia Ministry of Health | <https://www2.gov.bc.ca/gov/content/health/practitioner-professional-resources/bc-guidelines> |
| Canadian Medical Association (CMA) | <https://joulecma.ca/cpg/homepage> |
| Canadian Partnership Against Cancer | <https://www.partnershipagainstcancer.ca/tools/cancer-guidelines-database/> |
| Canadian Standards Association (CSA) | <https://store.csagroup.org/?cclcl=en_US> |
| The College of Physicians and Surgeons of Ontario (CPSO) | <https://www.cpso.on.ca/> |
| Ontario Association of Medical Laboratories (OAML) | <https://oaml.com/guidelines/> |
| Public Health Agency of Canada (PHAC) | <https://www.canada.ca/en/public-health/services/reports-publications/disease-prevention-control-guidelines.html> |
| Registered Nurses' Association of Ontario (RNAO) | <https://rnao.ca/bpg> |
| Winnipeg Regional Health Authority (WRHA) | <https://professionals.wrha.mb.ca/old/extranet/eipt/> |
| American Association for Clinical Chemistry (AACC) | <https://www.aacc.org/science-and-research/practice-guidelines> |
| Centers for Disease Control and Prevention (CDC) | <https://phgkb.cdc.gov/PHGKB/phgHome.action?action=home> |
| The Regulation and Quality Improvement Authority (RQIA) | <https://rqia.org.uk/what-we-do/rqia-s-funding-programme/guidelines/> |
| Haute Autorité de santé/ French National Authority for Health (HAS) | <https://www.has-sante.fr/jcms/c_6056/en/recherche-avancee?portlet=c_39085&search_antidot=&lang=en&typesf=guidelines> |
| Institute for Clinical Systems Improvement (ICSI) | <https://www.icsi.org/guidelines/> |
| National Health and Medical Research Council (NHMRC) | <https://www.clinicalguidelines.gov.au/> |
| National Institute for Health and Care Excellence (NICE) | <https://www.nice.org.uk/guidance/published> |
| Scottish Intercollegiate Guidelines Network (SIGN) | <https://www.sign.ac.uk/> |
| **Databases (free)** |  |
| Bandolier | <http://www.bandolier.org.uk/booth/booths/smoking.html> |
| LILACS | <https://lilacs.bvsalud.org/en/> |
| McMaster University, McMaster Health Forum | <https://www.healthsystemsevidence.org/> |
| NCBI | <https://www.ncbi.nlm.nih.gov/books> |
| TRIP Database | <https://www.tripdatabase.com/> |
| University of York (CRD) | <https://www.crd.york.ac.uk/CRDWeb/> |
| University of York. PROSPERO | <https://www.crd.york.ac.uk/prospero/> |
| US National Library of Medicine (NLM) | <http://www.ncbi.nlm.nih.gov/pubmed> |
| US National Library of Medicine & National Institutes of Health (NIH) | <http://www.ncbi.nlm.nih.gov/pmc/> |
| **Internet Search** |  |
| Google (first 5 pages) | [http://www.google.com](http://www.google.com/) |
| Google Scholar (first 5 pages) | <https://scholar.google.com/> |
| CMA | <https://www.cma.ca/about-cma> |
