# Supplementary material for: Screening for hypertension in adults: protocol for evidence reviews to inform a Canadian Task Force on Preventive Health Care guideline update
Source: Syst Rev. 2024 Jan 5;13:17. doi: 10.1186/s13643-023-02392-1 (PMC10768239; doi:10.1186/s13643-023-02392-1)
Supplement: Supplementary file 7 — Additional file 7. Draft data extraction items. [file 13643_2023_2392_MOESM7_ESM.docx]

## **Additional file 7: Draft data extraction items**

Study characteristics

- Authors (all KQs)
- Language (all KQs)
- Year of publication (all KQs)
- Country of study conduct (all KQs)
- Conflicts of interest (all KQs)
- Number of centres [if applicable] (KQ1, KQ2, KQ4)
- Study settings and location (KQ1,KQ2, KQ4)
- Study design (KQ1,KQ2, KQ4) or study designs included in the review (KQ3)
- Study designs included in review (KQ3)
- Duration of follow-up (all KQs)
- Publication type (all KQs)
- Objectives (all KQs)
- Funding (all KQs)
- Sample size (all KQs)
- Sampling technique (KQ1, KQ2, KQ4)
- Sample size calculation (KQ1, KQ2, KQ4)
- Study start and end date (KQ1, KQ2, KQ4)

Number of studies (KQ3)

- Protocol deviations (all KQs)

Search Details (KQ3)

- Number of databases searched
- Number of databases searched
- Data ranges of databases searched
- Language and publication restrictions

Population characteristics (all KQs)

- Recruitment method
- Randomization method
- Eligibility criteria for participants
- Response rate
- Information regarding respondent bias/representativeness of the included population
- Baseline demographic and clinical characteristics (e.g., age, sex, gender, socioeconomic status, geographical location, ethnic group, co-morbidities, screening history, family history, immigrant status)
- Number enrolled, assigned to each arm, received intended screening, analyzed, and losses and exclusions after randomization

Details about intervention/exposure

- Screening details (e.g., screening interval, universal/targeted, coverage, setting) (KQ1, KQ2)
- Type of screening test performed, method of collection (KQ1, KQ2)
- Threshold for discussion or diagnosis of hypertension (KQ3)
- Follow-up tests, procedures, care, or treatment (all KQs)
- Information presented to participants (KQ4)

Details about comparator

- Details on alternate type of screening performed, method of collection (KQ2)
- Threshold for discussion or diagnosis of hypertension (KQ3)
- Definition of no screening or usual care (KQ4)

Outcomes of interest (see Table 3-6 for KQ specific outcomes)

- Definition
- Measurement methods
- Ascertainment methods
- Timing

Analysis (All KQs)

- Unit of analysis
- Statistical methods and additional analyses (adjusted analyses, subgroup analyses, missing data)

Results (All KQs)

- Number of events
- Measures of effect (adjusted and/or unadjusted) and precision (e.g., 95% confidence interval)
- Measures of variability
- Cluster correlation coefficients (where relevant)
- Risk of Bias assessments for primary studies (KQ3)
- GRADE assessment for overall certainty and individual domains from reviews (KQ3)
