# Supplementary material for: Screening for hypertension in adults: protocol for evidence reviews to inform a Canadian Task Force on Preventive Health Care guideline update
Source: Syst Rev. 2024 Jan 5;13:17. doi: 10.1186/s13643-023-02392-1 (PMC10768239; doi:10.1186/s13643-023-02392-1)
Supplement: Supplementary file 8 — Additional file 8. UK NICE grading the strength of the body of evidence. [file 13643_2023_2392_MOESM8_ESM.docx]

## **Additional file 8: UK NICE grading the strength of the body of evidence**

**Citation**: 1. National Institute for Health and Care Excellence. Developing NICE guidelines: the manual (PMG20). Published October 2014. Accessed January 18, 2022. https://www.nice.org.uk/process/pmg20/resources/developing-nice-guidelines-the-manual-pdf-72286708700869

GRADE and GRADE-CERQual assess the certainty or confidence in the review findings by looking at features of the evidence found for each 'critical' and 'important' outcome or theme. GRADE is summarised in box 6.1 […]:

GRADE approach to assessing the certainty of evidence for intervention studies GRADE assesses the following features for the evidence found for each outcome:

• study limitations (risk of bias) – the internal validity of the evidence

• inconsistency – the heterogeneity or variability in the estimates of treatment effect across studies

• indirectness – the extent of differences between the population, intervention, comparator for the intervention and outcome of interest across studies

• imprecision – the extent to which confidence in the effect estimate is adequate to support a particular decision

• other considerations – publication bias, the degree of selective publication of studies.

[…] The certainty or confidence of evidence is classified as high, moderate, low or very low. In the context of NICE guidelines, it can be interpreted as follows:

• High – further research is very unlikely to change our recommendation.

Moderate – further research is likely to have an important impact on our confidence in the estimate of effect and may change the strength of our recommendation.

• Low – further research is very likely to have an important impact on our confidence in the estimate of effect and is likely to change the recommendation.

• Very low – any estimate of effect is very uncertain and further research will probably change the recommendation.

The approach taken by NICE differs from the standard GRADE and GRADE-CERQual system in 2 ways:

• it also integrates a review of the quality of cost-effectiveness studies (see the chapter on incorporating economic evaluation)

• it does not use 'overall summary' labels for the quality of the evidence across all outcomes or for the strength of a recommendation, but uses the wording of recommendations to reflect the strength of the evidence (see the chapter on writing the guideline).
