# Supplementary material for: Screening for hypertension in adults: protocol for evidence reviews to inform a Canadian Task Force on Preventive Health Care guideline update
Source: Syst Rev. 2024 Jan 5;13:17. doi: 10.1186/s13643-023-02392-1 (PMC10768239; doi:10.1186/s13643-023-02392-1)
Supplement: Supplementary file 9 — Additional file 9. USPSTF grading the strength of the body of evidence. [file 13643_2023_2392_MOESM9_ESM.docx]

## **Additional file 9: USPSTF grading the strength of the body of evidence**

**Citation**: Guirguis-Blake JM, Evans CV, Webber EM, Coppola EL, Perdue LA, Weyrich MS (2020) Screening for Hypertension in Adults: A Systematic Evidence Review for the U.S. Preventive Services Task Force. <https://www.uspreventiveservicestaskforce.org/uspstf/document/draft-evidence-review/hypertension-in-adults-screening>.

We graded the strength of the overall body of evidence for each KQ. We adapted the Evidence-based Practice Center approach, ^1^ which is based on a system developed by the Grading of Recommendations Assessment, Development and Evaluation (GRADE) Working Group. ^2^ Our method explicitly addresses four of the five Evidence-based Practice Center-required domains: consistency (similarity of effect direction and size), precision (degree of certainty around an estimate), reporting bias (potential for bias related to publication, selective outcome reporting, or selective analysis reporting), and study quality (i.e., study limitations). We did not address the fifth required domain—directness—as it is implied in the structure of the KQs (i.e., pertains to whether the evidence links the interventions directly to a health outcome).

Consistency was rated as reasonably consistent, inconsistent, or not applicable (e.g., single study). Precision was rated as reasonably precise, imprecise, or not applicable (e.g., no evidence). Reporting bias was rated as suspected, undetected, or not applicable (e.g., when there is insufficient evidence for a particular outcome). Study quality reflects the quality ratings of the individual studies and indicates the degree to which the included studies for a given outcome have a high likelihood of adequate protection against bias. The body-of-evidence limitations field highlights important restrictions in answering the overall KQ.

We graded the overall strength of evidence as high, moderate, or low. “High” indicates high confidence that the evidence reflects the true effect and that further research is very unlikely to change our confidence in the estimate of effects. “Moderate” suggests moderate confidence that the evidence reflects the true effect and that further research may change our confidence in the estimate of effect and may change the estimate. “Low” indicates low confidence that the evidence reflects the true effect and that further research is likely to change our confidence in the estimate of effect and is likely to change the estimate. A grade of “insufficient” indicates that evidence is either unavailable or does not permit estimate of an effect. Two independent reviewers rated each KQ according to consistency, precision, reporting bias, and overall strength of evidence grade. We resolved discrepancies through consensus discussion involving more reviewers.

**References**

1. Berkman N, Lohr K, Ansari M, et al. Grading the Strength of a Body of Evidence When Assessing Health Care Interventions for the Effective Health Care Program of the Agency for Healthcare Research and Quality: An Update. Methods Guide for Effectiveness and Comparative Effectiveness Reviews. AHRQ Publication No. 10(14)-EHC063-EF. Rockville, MD: Agency for Healthcare Research and Quality; 2014. p. 314-49. PMID.

2. Atkins D, Eccles M, Flottorp S, et al. Systems for grading the quality of evidence and the strength of recommendations I: critical appraisal of existing approaches The GRADE Working Group. BMC Health Serv Res. 2004;4(1):38. PMID: 15615589. <http://dx.doi.org/10.1186/1472-6963-4-38>
