# Supplementary material for: Screening for hypertension in adults: protocol for evidence reviews to inform a Canadian Task Force on Preventive Health Care guideline update
Source: Syst Rev. 2024 Jan 5;13:17. doi: 10.1186/s13643-023-02392-1 (PMC10768239; doi:10.1186/s13643-023-02392-1)
Supplement: Supplementary file 10 — Additional file 10. Stakeholder review and feedback. [file 13643_2023_2392_MOESM10_ESM.docx]

## Additional file 10. Stakeholder review and feedback

| **Section** | **Comment** | **Reviewer** | **Response** |
| --- | --- | --- | --- |
| N/A | I would just like to ensure that we do get a wide variety of populations as the data is reviewed and analyzed. Wondering if as part of the data summary, there could be a table showing exactly what populations/ethnicities were included across the studies reviewed. | Reviewer #1 | Thank you for your suggestion. We will present demographic information on the population (including race/ethnicities) in any included studies as part of our synthesis of study characteristics for each key question. |
| Table 3 | [Table 1 and 2 seem clear. Table 3 questions I have are whether or not there will be clear indications of whether the settings have “personnel trained in blood pressure measurements”. MD and NP and other nursing professionals (RN, LPN) would certainly have this but some offices may have medical secretaries completing BP checks. There staff may or may not be properly or professionally trained. Will this matter? Also wondering why only “English” for this while the others are English and French. | Reviewer #1 | Thank you. We acknowledge that it may be unclear if a particular study reported if the setting included those trained in blood pressure measurement. We are relying on the 2021 review by the USPSTF, so will assume that any included studies met this criteria. In our manuscript discussion we will note that in real life clinical scenarios there may be individuals with varying levels of training.  The USPSTF review excluded any studies published in a language other than English. We will review French studies excluded for this reason at the full-text stage and include these studies if they meet our other eligibility criteria for KQ1. |
| Table 3 | According to Table 3, the population of interest includes all individuals aged 18 years and older with the exclusion of pregnant women, children and youth less than 18 years, persons in institutions, secondary hypertension cases as well as highly selected groups of people. Although some at-risk populations are mentioned in the background section (e.g. Black and South Asian individuals and Indigenous people), it is unclear whether the search strategy will be targeting those groups specifically and if recommendations will target some of those groups. Also, will the guidelines specify recommendations for rural and remote populations that have limited access to care? Are there any lessons learned from the pandemic (e.g. virtual care) that could be explored? | Reviewer #2 | Thank you for your comments. We agree that it is important to capture information for these high-risk populations. Subgroups of interest include those in different settings, socioeconomic levels and area of residence. We intend to synthesize the information for these populations in a subgroup analysis to inform Task Force recommendations. Considerations related to access and health equity will be also explored by the Task Force in their evidence-to-decision framework. |
| N/A | ​​[Will macrovascular CVD events also include transient ischemic attacks or only acute stroke? It should be considered. It is included in the stroke guidelines, see link listed under question 4. ]​ | Reviewer #2 | Thank you. The outcomes under macrovascular complications are examples and do not constitute an exhaustive list of macrovascular outcomes. We will also consider other common macrovascular outcomes, such as transient ischemic attacks or cognitive impairment. |
| N/A | ​​Consider reviewing Diabetes Canada Clinical Practice Guidelines and Canadian Stroke Best Practices. https://guidelines.diabetes.ca/cpg https://www.strokebestpractices.ca/recommendations?_ga=2.96400604.1556656657.1637277246-1003820302.1637277246​ | Reviewer #2 | Thank you for the suggestion. We will add this to our list of sources to be screened in our grey literature search. |
| N/A | ​​No major concerns, but it would be important to clarify whether the Task Force intends to develop similar guidelines for children and youth and how its work aligns with that of Hypertension Canada. I understand that new guidelines were released by Hypertension in Canada in 2020 and new screening thresholds impact the epidemiology of hypertension greatly in children and youth according to this recently published paper:  https://www150.statcan.gc.ca/n1/pub/82-003-x/2023004/article/00001-eng.htm. Further, given the different screening thresholds for individuals with diabetes and the high prevalence of comorbid hypertension and diabetes, should one of the research questions focus specifically on that population? For instance, are CVD outcomes the same in that subpopulation or are they at an even higher risk than those without diabetes?  ​ | Reviewer #2 | Thank you. The current evidence review is meant to inform the guidelines pertaining to screening for hypertension in adults. We agree that it is important to also consider guidelines for children and adolescents and will bring this forward to the topics working group.  We acknowledge that there is a high degree of comorbidity for individuals with hypertension and diabetes and will collect any data on the proportion of the population with diabetes, if reported. If possible, we will explore differential effects of screening in this population through subgroup analyses. |
| **Table 1** Key questions to inform an update of recommendations by the Task Force on hypertension screening in adults aged 18 years and older in primary care | One of the key questions missing here is the predisposition of certain races to earlier HTN, worse outcomes. Knowledge of this within primary care is important as Canada's population diversifies. | Reviewer #3 | Thank you for your feedback. We agree that it is important to capture information for high-risk populations, including those related to race/ethnicity. When possible for each key question, we intend to capture synthesize the information for these populations in a subgroup analysis to inform Task Force recommendations. |
|  | US has had recommendation that is lower than both in Canada and UK. Having one universal threshold is better. Are there studies that show the lower threshold in US has any CVD benefits? | Reviewer #3 | Thank you. We intend to review the available evidence on different blood pressure thresholds for treatment initiation to inform our third key question on differing thresholds for treatment initiation discussions. |
| N/A | Consider including (where possible) a sub-analysis of Inuit populations, as they are quite distinct from other Indigenous populations | Reviewer #4 | Thank you for the suggestion. We will separate any data regarding Indigenous People in Canada, it should be separated in a distinctions-based way (First Nation, Métis, and Inuit) if available within the reviewed data sets. |
| N/A | Consider, where possible, clarifying what an “appropriate” visit for HTN screening is – this has not always been well described in previous guidelines and recommendations | Reviewer #4 | Thank you. While we do not use the term “appropriate visits” in our evidence review, we have acknowledged that this is the terminology used in the prior 2012 recommendations by the Task Force. We will clarify any language surrounding “appropriate” visits in our evidence review. |
| N/A | KQ3 – the focus on treatment initiation may, technically speaking, lead to exclusion of many of the key studies that inform BP treatment thresholds.  Many of the major trials (like SPRINT and even older ones like HOT) did not randomize people to different initiation thresholds but rather to different treatment targets (and they all had to have SBP > 130 to enter study).  You might consider breaking KQ3 into two or more parts – it is currently asking several questions each of which is a pretty big question in its own right (ie – about treatment thresholds, the impact of high CV risk on treatment threshold, and treatment thresholds according to BP measurement method).   It is not clear if lifestyle change/advice is one of the treatments being considered.  In my opinion it will be hard to operationalize the phrase “initiate discussions”.  In reality all treatments (ideally) follow a discussion – I am not sure there is a clear enough gap between initiating discussions and prescribing an antihypertensive that you wouldn’t just say “initiate treatment” or “offer treatment”. | Reviewer #5 | Thank you for the feedback. We recognize that some of these major trials may be excluded. The goal of this key question is to examine clinical blood pressure thresholds for initiating treatment (or discussions surrounding treatment initiation). While trials comparing different treatment targets may be relevant for deciding on the specific treatment plan, this is out of scope for the current guideline. Instead, the intent is to decide at what level of blood pressure should treatment be considered.  The language surrounding “discussions of treatment” vs. “treatment initiation” is alluding to the process of shared decision making. The CTFPHC does not intend to recommend thresholds for treatment initiation, but rather, only to begin a process of discussing different methods of treatment. |
| N/A | KQ2 – I understand why the language focuses on patients without a dx of HTN, but I don’t think you are going to find too much specifically in patients without diagnosed hypertension.  I am also not sure there is a clear reason to think BP measurement technique accuracy should differ in people without vs with HTN. | Reviewer #5 | Thank you for your suggestion. We agree that it is potentially unlikely that we will find evidence specifically in patients without a diagnosis of hypertension. We have therefore added the criteria to note that we will consider evidence in patients with documented hypertension, if we fail to find such evidence. |
| N/A | ​​Mostly the questions address important issues.  However, I am not sure KQ4 is as clinically applicable.  I have been practicing primary care for nearly twenty years – it is very difficult to imagine the practicality of holding shared making discussions with every patient before taking their vitals.  On the other hand, it is really important for us to understand how different approaches to BP measurement compare.  There are questions related to this that would be useful to consider.  For instance, almost all the information we have about when and how aggressively to treat hypertension comes from trials in which BP was measured using “research quality” blood pressures (for a number of influential studies this was the average of three automated BP cuff measures in a quiet room, unattended).  It would be useful to know how different approaches (HBPM, ABPM, typical clinic BPs) compare to “research quality” measures.  When during a clinic visit should the BP be measured?  Is a wrist cuff any good?  In short, we do an awful job of measuring BP and a lot of the challenge in diagnosing and managing HTN has to do with measurement issues.  Guidance about technique, accuracy, timing etc would be helpful to clinicians, particularly since HBPM has become more widespread.  ​ | Reviewer #5 | Thank you for your feedback. The goal of our key question on patient values and preferences (KQ4) for screening is to capture and synthesize information to inform the Task Force on any literature relating to the patient perspective on hypertension screening, as part of the evidence review. This will aid the Task Force in weighing patient perspectives while writing their recommendations.  We agree that there are several other important clinical questions regarding blood pressure measurement and implementation in clinical practice. Evidence on the association between different blood pressure measurement methods and cardiovascular outcomes will be captured by our second key question. As screening is the primary focus of this guideline, other questions on the diagnostic accuracy of different blood pressure measurement methods are, unfortunately, out-of-scope for this evidence review. However, we will bring forth these concerns to the Task Force to consider these issues when formulating their recommendations. |
| N/A | No concern on specific populations of interest. To ensure accurate representation, if data is presented regarding Indigenous People in Canada, it should be separated in a distinctions-based way (First Nation, Métis, and Inuit) if available within the reviewed data sets. To further gender-based equity, please change the word ‘himself’ to ‘themselves’ in the last paragraph, last sentence of the “Screening for Hypertension” section on page 13. | Reviewer #6 | Thank you for the suggestion. We will separate any data regarding Indigenous People in Canada, it should be separated in a distinctions-based way (First Nation, Métis, and Inuit) if available within the reviewed data sets. We have also made the wording change, as you suggested. |
| Table 3 | Some sections mention specifically “noninvasive brachial blood pressure measurement” while others do not. I would suggest consistency in this description.    Table 3 Outcomes mentions “vascular dementia” in microvascular outcomes. This does not appear elsewhere where microvascular outcomes are mentioned (“eg renal disease, retinal disease”). I would suggest consistency here. | Reviewer #7 | Thank you for this suggestion. We will update the eligibility criteria to ensure that the descriptions are consistent between key questions, as appropriate.  The outcomes under microvascular complications are examples and do not constitute an exhaustive list of microvascular outcomes. We will also consider other common microvascular outcomes, such as vascular dementia. |
